# WNK1 is a chloride-stimulated scaffold that regulates mTORC2 activity and ion transport

**DOI:** 10.1242/jcs.260313

**Published:** 2022-12-05

**Authors:** Bidisha Saha, Deise C. A. Leite-Dellova, John Demko, Mads Vaarby Sørensen, Enzo Takagi, Catherine E. Gleason, Waheed Shabbir, David Pearce

**Affiliations:** ^1^ Division of Nephrology, Departments of Medicine and Cellular & Molecular Pharmacology, University of California at San Francisco, San Francisco, CA 94158, USA; ^2^ Department of Veterinary Medicine, Faculty of Animal Science and Food Engineering, University of São Paulo, Pirassununga, Sao Paulo 13635-900, Brazil; ^3^ Departments of Biomedicine and Physiology, Aarhus University, 8000 Aarhus C, Denmark

**Keywords:** mTOR, mTORC2, Rictor, SGK1, WNK1, WNK kinase, ENaC, Electrolytes, Ion transport, K^+^ homeostasis, Kidney epithelial cells, Protein–protein interaction

## Abstract

Mammalian (or mechanistic) target of rapamycin complex 2 (mTORC2) is a kinase complex that targets predominantly Akt family proteins, SGK1 and protein kinase C (PKC), and has well-characterized roles in mediating hormone and growth factor effects on a wide array of cellular processes. Recent evidence suggests that mTORC2 is also directly stimulated in renal tubule cells by increased extracellular K^+^ concentration, leading to activation of the Na^+^ channel, ENaC, and increasing the electrical driving force for K^+^ secretion. We identify here a signaling mechanism for this local effect of K^+^. We show that an increase in extracellular [K^+^] leads to a rise in intracellular chloride (Cl^−^), which stimulates a previously unknown scaffolding activity of the protein ‘with no lysine-1’ (WNK1) kinase. WNK1 interacts selectively with SGK1 and recruits it to mTORC2, resulting in enhanced SGK1 phosphorylation and SGK1-dependent activation of ENaC. This scaffolding effect of WNK1 is independent of its own kinase activity and does not cause a generalized stimulation of mTORC2 kinase activity. These findings establish a novel WNK1-dependent regulatory mechanism that harnesses mTORC2 kinase activity selectively toward SGK1 to control epithelial ion transport and electrolyte homeostasis.

## Teaser

**Summary:** mTORC2 is a master regulator of multiple signaling pathways; however, its physiological roles are poorly characterized. Here, we identify a surprising new mechanism for its role in electrolyte homeostasis.

## INTRODUCTION

Mammalian (or mechanistic) target of rapamycin (mTOR) is well recognized as a central controller of cell growth and metabolism (Liu and Sabatini, 2020). It is found in two structurally and functionally distinct multiprotein complexes, mTORC1 and mTORC2 (Fu and Hall, 2020; Loewith et al., 2002; Sarbassov et al., 2004). mTORC1 includes the defining core component, Raptor, and plays central roles in growth, tissue remodeling and immune function by regulating ribosome activity through phosphorylation of p70-S6 kinase and 4-EBP (also known as RPS6KB1 and EIF4EBP1, respectively), among others, in a rapamycin-sensitive manner (Fu and Hall, 2020). mTORC2 is defined by two core subunits, rapamycin-insensitive companion of TOR (Rictor) and mammalian stress-activated protein kinase-interacting protein 1 (SIN1; also known as mSin1 and MAPKAP1), which together control mTORC2 assembly and substrate specificity, in particular targeting the AGC kinases, Akt family proteins (hereafter Akt), SGK1 and protein kinase C (PKC) (Fu and Hall, 2020). It was originally identified as a rapamycin-resistant kinase complex involved in growth control and actin cytoskeleton regulation through phosphorylation of Akt and PKC at a conserved site termed the ‘hydrophobic motif’ (HM) (Jacinto et al., 2004; Sarbassov et al., 2005a,b). HM sequence homology led to studies demonstrating similar mTORC2-dependent phosphorylation of SGK1 at Ser-422, and that it has central roles in electrolyte homeostasis (Garcia-Martinez and Alessi, 2008; Gleason et al., 2015; Grahammer et al., 2016; Lu et al., 2010).

The roles of mTORC2 in responding to hormones, growth factors and cytokines to regulate cell growth, ion transport and metabolism have been extensively investigated; however, recent studies also point to roles in rapid cell autonomous responses to physiological stresses, such as ambient temperature and blood K^+^ levels (Allu et al., 2021; Fu and Hall, 2020; Sørensen et al., 2019). One recent report supported the idea that local extracellular K^+^ concentration rapidly stimulates mTORC2-dependent phosphorylation of SGK1, with downstream effects on the epithelial Na^+^ channel ENaC (Sørensen et al., 2019). This signaling module was further shown to be central to rapid stimulation of K^+^ secretion, consistent with the well-established role of ENaC in controlling the electrical gradient driving apical K^+^ transport, particularly in the context of rapid responses to K^+^ (Frindt and Palmer, 2009). Interestingly, the serine-threonine kinase WNK1 has been shown to potently stimulate mTORC2 activity through a non-catalytic mechanism; however, the functional consequences and underlying mechanism had not been examined (Sørensen et al., 2019). Although most studies have focused on kinase-dependent roles of WNKs in regulating SPAK (also known as STK39) and OSR1 and hence cation-chloride cotransporters such as NCC (SLC12A3) and NKCC1 (SLC12A2) and NKCC2 (SLC12A1) (Murillo-de-Ozores et al., 2020; Terker et al., 2016), a previous report identified non-catalytic stimulation of SGK1 and ENaC (Xu et al., 2005b). Similarly, WNK1-mediated activation of BK channels through non-catalytic mechanisms has been described (Webb et al., 2016). However, the role of mTORC2 was unknown, and the underlying signaling mechanisms for non-canonical signaling have yet to be investigated.

To further explore the mechanism and functional implications of WNK1-stimulated mTORC2 activity, we examined SGK1 phosphorylation and its downstream effects on ENaC activity in mTORC2- and WNK1-deficient renal epithelial cells. Additionally, based on observations that Cl^–^ can bind WNK1 (Piala et al., 2014) and inhibit its catalytic activity in a K^+^-regulated fashion (Penton et al., 2016; Terker et al., 2015) we also examined the role of Cl^–^ in controlling non-catalytic effects of WNK1. Our data support the idea that WNK1 is a Cl^–^-regulated scaffold for mTORC2-mediated SGK1 phosphorylation, and thereby stimulates ENaC-dependent Na^+^ transport. This mechanism represents a new mode of mTORC2 kinase signaling through which local extracellular K^+^ concentration selectively activates one of the substrates of mTORC2 (SGK1), but not others (Akt and PKC) to regulate ion transport and electrolyte homeostasis.

## RESULTS

### WNK1 is required for K^+^-stimulated SGK1 phosphorylation and ENaC activation in kidney collecting duct cells

**Fig. 1.**
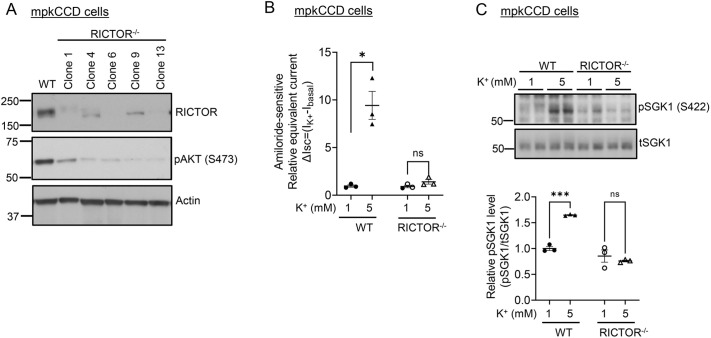
**Extracellular K^+^ stimulates ENaC activity through mTORC2–SGK1 signaling in cultured CCD cells.** (A) Generation of mTORC2-deficient mpkCCD cells. Cells were subjected to CRISPR/Cas9-mediated *RICTOR* gene deletion as described in the Materials and Methods. Multiple clones were obtained and characterized for Rictor expression and the ability to generate high-electrical resistance monolayers on Transwell filters. Results are representatives of *n*=2 biological replicates. (B,C) Effect of extracellular K^+^ on ENaC current (B) and SGK1 HM phosphorylation (C) in WT versus RICTOR^−/−^ (clone 6) mpkCCD cells. Cells were adapted to 1 mM [K^+^] for 3 h, medium [K^+^] was increased by addition of KCl, and incubated for 1 h additional prior to measurement of amiloride-sensitive current (B). Cells were then lysed and prepared for western blotting, and stained with antibodies as indicated (C). (C, upper panel) Western blot images showing blots stained with anti-phospho (p)SGK1 S422 (sc-16745, Santa Cruz Biotechnology), and total (t)SGK1 (5188, Sigma) antibodies. (C, lower panel) Quantification of phospho-SGK1/total SGK1 (as described in the Materials and Methods). In B and C, data are mean±s.e.m. from *n*=3 biological replicates. *P<0.05; ***P<0.001; ns, not significant [multiple unpaired two-tailed Student's *t-*test (B); two-way ANOVA with Bonferroni's multiple-comparison test (C)].

**Fig. 2.**
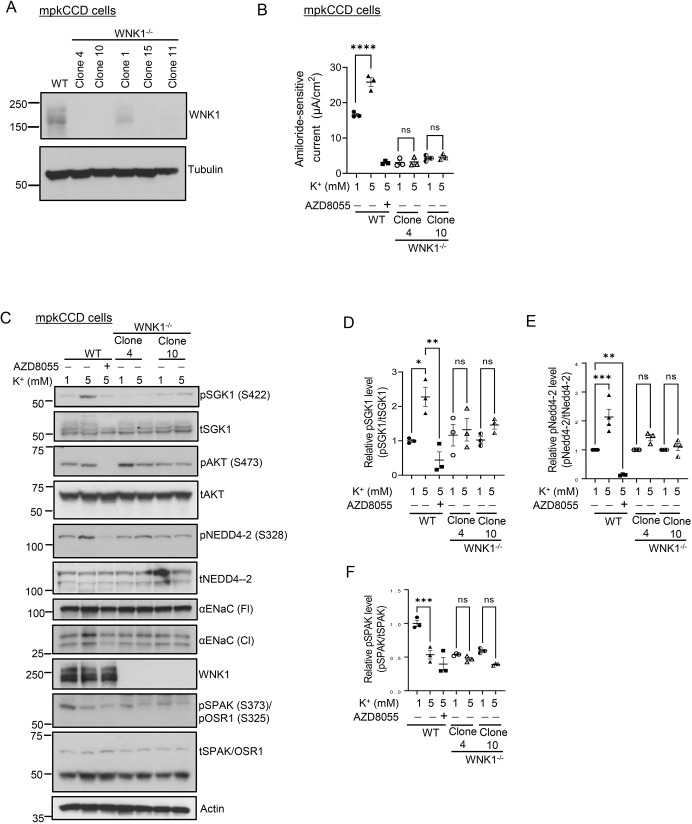
**WNK1 is required for K^+^-induced ENaC activity and SGK1 phosphorylation.** (A) Western blot analyses of WNK1 protein in WT and WNK1^−/−^ mpkCCD clones. WNK1 was deleted from mpkCCD cells using CRISPR/Cas9 (see Materials and Methods for details). WNK1 protein was detected using anti-WNK1 antibody. Results are representatives of *n*=2 biological replicates. (B) Effect of extracellular K^+^ on ENaC current in WT and WNK1^−/−^ mpkCCD cells. WT and KO cells (two independent clones) were grown on Transwell filters, adapted to 1 mM [K^+^] on the basolateral side, and then the medium [K^+^] was either raised to 5 mM or kept at 1 mM for 1 h prior to measurement of amiloride-sensitive current. All values are means±s.e.m. from *n*=3 biological replicates. µA, microamperes. AZD8055, mTOR inhibitor. *n*=3 independent experiments. (C) Effect of extracellular K^+^ on SGK1 phosphorylation in WT and WNK1^−/−^ mpkCCD cells. Cells as in B, were harvested and subjected to western blotting for various downstream targets of mTORC2 and WNK1 as indicated. Fl, full length; Cl, cleaved; t, total. pSGK1 and WNK1 proteins were detected using anti-phospho-SGK1 S422 (sc-16745, Santa Cruz Biotechnology), and anti-WNK1 (AF2849, R&D Systems) antibody, respectively. Results are representatives of *n*=3 biological replicates. (D–F) Quantification of bands in western blots from C. Note that stimulatory effects of 5 mM [K^+^] on pSGK1 (direct substrate), and pNEDD4-2 (SGK1 substrate) are observed in WT but not in WNK1-deleted cells. Phospho-SPAK was inhibited by 5 mM [K^+^] in WT cells but not in WNK1^−/−^ cells consistent with prior literature (see text for details). All values are means±s.e.m. from *n*=3 biological replicates. *P<0.05, **P<0.01, ***P<0.001, ****P<0.0001; ns, not significant (one-way ANOVA with Bonferroni's multiple-comparison test).

As a first step toward characterizing the relationship between mTORC2 and WNK1 in K^+^-induced ENaC activation, we used mpkCCD cells derived from mouse kidney cortical collecting duct (Bens et al., 1999). We generated mTORC2-deficient cells using CRISPR/Cas9 to delete Rictor (Fig. 1A). As shown previously (Sørensen et al., 2019), wild-type (WT) mpkCCD cells demonstrated robust K^+^-stimulated ENaC current and SGK1 Ser-422 phosphorylation (Fig. 1B,C, respectively). Consistent with prior studies in which mTOR was pharmaceutically inhibited or knocked down through RNAi (Lu et al., 2010; Sørensen et al., 2019), RICTOR^−/−^ mpkCCD cells showed a marked decrease in both ENaC currents and SGK1 Ser-422 phosphorylation (pSGK1), and showed no response to a change in the medium [K^+^] (Fig. 1B,C). Based on the phenotype of familial hyperkalemia and hypertension (FHHt) (Wilson et al., 2001), and a report that WNK1 could stimulate SGK1 and ENaC (Xu et al., 2005a), we next generated and characterized WNK1^−/−^ mpkCCD cells, also using the CRISPR/Cas9 system (Fig. 2A). Baseline ENaC currents were reduced in WNK1^−/−^ cells (Fig. S1A,B), and K^+^-induced activation was abolished (Fig. 2B); pSGK1 and SGK1-dependent phosphorylation of Nedd4-2 (also known as NEDD4L) were also reduced and K^+^-stimulated phosphorylation was lost (Fig. 2C–E; Fig. S2). Expression of active processed form of αENaC (encoded by *SCNN1A*) (cleaved αENaC) was increased in K^+^-stimulated WT mpkCCD cells but not in WNK1^−/−^ cells [Fig. 2C, ENaC (Cl)]. It is notable that phosphorylated Akt was also detected (Fig. 2C, pAKT) in WT mpkCCD cells; however, it was not induced by increased K^+^. Interestingly, Akt HM phosphorylation was unaffected by loss of WNK1 (Fig. 2C, pAKT, lanes 4–6), consistent with previous results in HEK-293 cells (Sørensen et al., 2019). Finally, WNK-dependent SPAK phosphorylation was inhibited by elevated [K^+^] in WT mpkCCD cells, and this activity was attenuated in WNK1^−/−^ cells, and K^+^ regulation was lost (Fig. 2F), as is well established in renal distal convoluted tubule (DCT) cells (Sorensen et al., 2013). Thus, WNK1-deficient mpkCCD cells have severe defects in both mTORC2–SGK1 and canonical WNK–SPAK signaling.

### WNK1 kinase activity is not required for SGK1 phosphorylation or ENaC stimulation

**Fig. 3.**
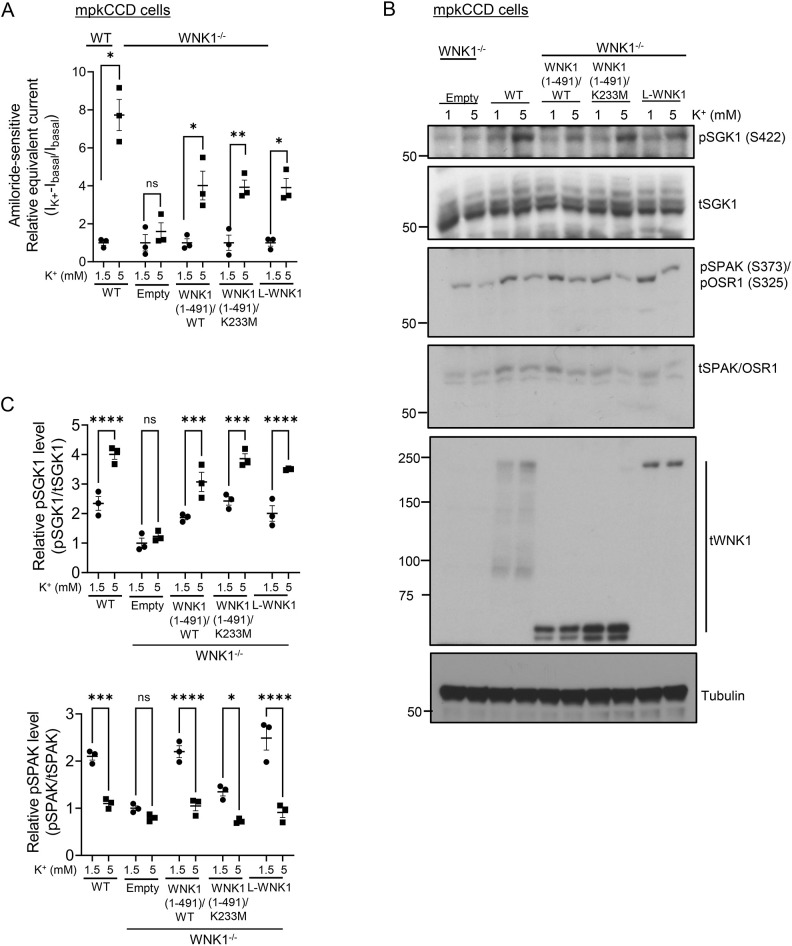
**WNK1 mediates K^+^-stimulated SGK1 phosphorylation and ENaC activation through a non-catalytic mechanism in mpkCCD cells.** (A) Transfected kinase-active WNK1 (aa 1–491 or full-length L-WNK1) or kinase-dead WNK1 mutant (aa 1–491) (K233M) all similarly restored extracellular K^+^-stimulation of ENaC activity in mpkCCD cells. WNK1-deficient mpkCCD cells were transfected with empty vector, WT WNK1 (1–491), WNK1 (1–491)/K233M or WT L-WNK1, and cells were treated as in Fig. 2C. In WNK1-deficient cells transfected with empty vector (empty), raising extracellular [K^+^] to 5 mM failed to stimulate ENaC activity. ‘WT’ shows currents in untransfected WT mpkCCD cells. Data represent fold increase in amiloride-sensitive current relative to baseline in 1 mM K^+^. All values are means±s.e.m. from *n*=3 biological replicates. (B) Representative western blot showing the effect of WT (1–491 or full-length L-WNK1) and kinase-dead WNK1 mutant (1–491) on SGK1 and SPAK phosphorylation in response to changes in extracellular [K^+^] in transfected WNK1^−/−^ mpkCCD cells from A. pSGK1 and WNK1 proteins were detected using anti-phospho-SGK1 S422 (sc-16745, Santa Cruz Biotechnology), and anti-WNK1 (NBP2-75712, Novus Biologicals) antibodies, respectively. t, total. Results are representatives of *n*=3 biological replicates. (C) Quantification of pSGK1 (upper panel) and pSPAK/OSR1 (lower panel) bands in western blots from B. All values are means±s.e.m. from *n*=3 biological replicates. *P<0.05; **P<0.01; ***P<0.001; ****P<0.0001; ns, not significant [multiple unpaired two-tailed Student's *t*-test (A); one-way ANOVA with Bonferroni's multiple-comparison test (C)].

**Fig. 4.**
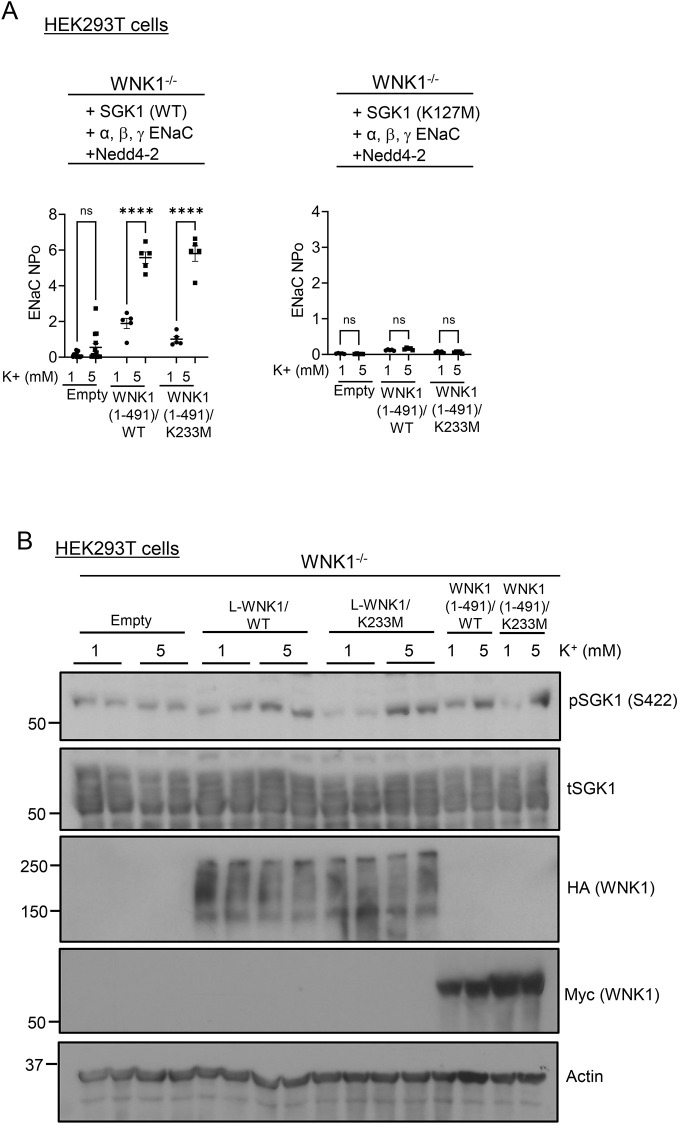
**The kinase activity of WNK1 is not required for K^+^-stimulated SGK1 phosphorylation and ENaC activity in HEK-293 cells.** (A) Single-channel ENaC current measured by patch-clamp in WNK1^−/−^ HEK-293 cells, showing the effect of WT and kinase-dead WNK1 on K^+^-stimulated ENaC activity. Cells were transfected with ENaC, Nedd4-2, Myc-tagged WT (aa 1–491) or kinase-dead (aa 1–491)/K233M WNK1, and WT SGK1 (left panel) or kinase-dead SGK1 (K127M) (right panel). Cells were adapted to 1 mM [K^+^], and then bath [K^+^] was either raised to 5 mM or kept at 1 mM for 1 h prior to measurement of amiloride-sensitive current by patch-clamp. Note that extracellular K^+^ stimulated ENaC current in cells expressing either WT or kinase-dead WNK1 and WT SGK1. However, in cells expressing kinase-dead SGK1 (K127M), raising extracellular [K^+^] to 5 mM failed to stimulate ENaC current. All values are means±s.e.m.; *n*=5 in each group. ****P<0.001 (two-way ANOVA with Bonferroni's multiple-comparison test). (B) Western blot showing the effect of WT and kinase-dead full length (L-WNK1) and truncated (WNK1 1–491) WNK1 on SGK1 phosphorylation. Kinase active (WT) and kinase-dead (K233M) forms similarly stimulated pSGK1 (S422). WNK1-deficient HEK-293 cells were transfected with empty vector, WT HA-L-WNK1 or kinase-dead HA–L-WNK1/K233M and WT Myc–WNK1 (aa 1–491) or kinase-dead Myc–WNK1 (aa 1-491)/K233M along with WT SGK1. Cells were adapted to 1 mM extracellular [K^+^] and then transferred to 5 mM K^+^ media or maintained in 1 mM for 1 h prior to processed for western blot analysis. pSGK1 protein was detected using anti–phospho-SGK1 S422 (sc-16745, Santa Cruz Biotechnology) antibody. t, total. Results are representatives of *n*=3 biological replicates.

We next examined the role of WNK1 kinase activity on the mTORC2–SGK1 signaling module. First, we demonstrated that transfection of WNK1^−/−^ mpkCCD cells with WT WNK1 (both long WNK1 and a truncated form known to phosphorylate SPAK) restored K^+^-regulated ENaC activation (Fig. 3A) and SGK1 HM phosphorylation (Fig. 3B, pSGK1; Fig. 3C, upper panel). However, in striking contrast to SPAK regulation (Fig. 3B, pSPAK, pOSR1; Fig. 3C, lower panel), ENaC activation and SGK1 phosphorylation were restored as well by kinase-dead WNK1 (K233M) as it was by WT (Fig. 3A,B). Similar effects were seen in WNK1^−/−^ HEK-293 cells transfected with ENaC, SGK1 and Nedd4-2 (Fig. S3); ENaC activity (as measured by patch clamp) was nearly undetectable in these cells (Fig. 4A, left panel; Fig. S4) and was fully restored by either WT or kinase-dead WNK1. It is notable that the effects of K^+^ were abrogated in HEK-293 cells transfected with kinase-dead SGK1 (K127M), which had a dominant-negative effect on ENaC, as has been described previously (Fig. 4A, right panel) (Loffing et al., 2001), supporting a central role of SGK1 in these effects. Likewise, extracellular K^+^ stimulation of SGK1 phosphorylation was restored by WT and kinase-dead L-WNK1 and their truncated variants (Fig. 4B).

**Fig. 5.**
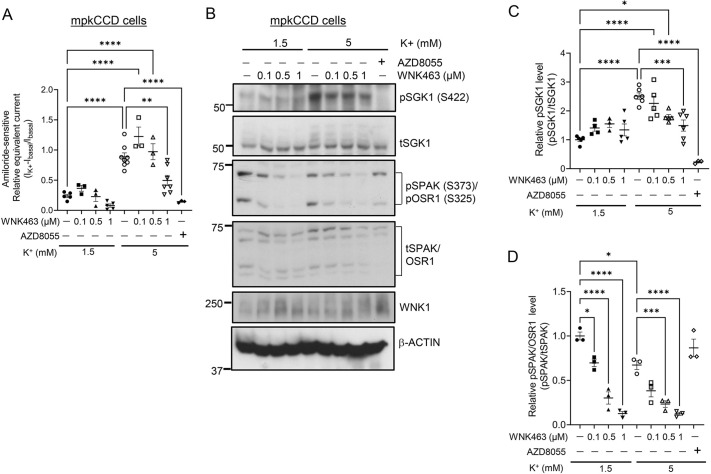
**Effect of the WNK kinase inhibitor WNK463 on K^+^-induced ENaC current and SGK1 phosphorylation in WT mpkCCD cells.** (A–D) mpkCCD cells were adapted to 1 mM extracellular [K^+^]. The WNK1 inhibitor WNK463 or vehicle was added at concentrations shown and extracellular [K^+^] was then increased by addition of KCl for 1 h prior to determining amiloride-sensitive Na^+^ current (A) as described in the Materials and Methods. Note that in last groups in A and C, and last lane in B, cells were treated with the mTOR inhibitor AZD8055. Cells were then lysed and prepared for western blotting, and stained with antibodies as indicated in labels for the representative blot in B. pSGK1 and WNK1 proteins were detected using anti-phospho-SGK1 S422 (sc-16745, Santa Cruz Biotechnology), and anti-WNK1 (AF2849, R&D Systems) antibody, respectively. t, total. Bands in western blots for pSGK1 and pSPAK/pOSR1 were quantitated by densitometry in C and D, respectively. All values are means±s.e.m. from from *n*=3 independent experiments. As noted, WNK463 inhibited pSPAK with an IC-50 of ∼100 nM. At high concentration (1 µM) WNK463 reduced SGK1 phosphorylation (see text for details). *P<0.05; **P<0.01; ***P<0.001; ****P<0.0001 (one-way ANOVA with Bonferroni's multiple-comparison test).

As an alternative approach to assessing whether WNK kinase activity was dispensable, we examined the effect of a selective WNK inhibitor, WNK463 (Yamada et al., 2016), on mTORC2-dependent SGK1 phosphorylation and ENaC activation. Consistent with the results in Figs 3 and 4, WNK463 had no significant effect on K^+^-induced ENaC current (Fig. 5A) or pSGK1 (Fig. 5B,C) at 100 or 500 nM, concentrations that markedly inhibit pSPAK (Fig. 5D). At 1 µM, WNK463 did have a significant effect on ENaC and pSGK1, possibly due to off-target inhibition of mTORC2 or inhibition of a less-sensitive WNK kinase isoform.

### K^+^-stimulated WNK1–mTORC2-mediated SGK1 phosphorylation is Cl^−^ dependent

**Fig. 6.**
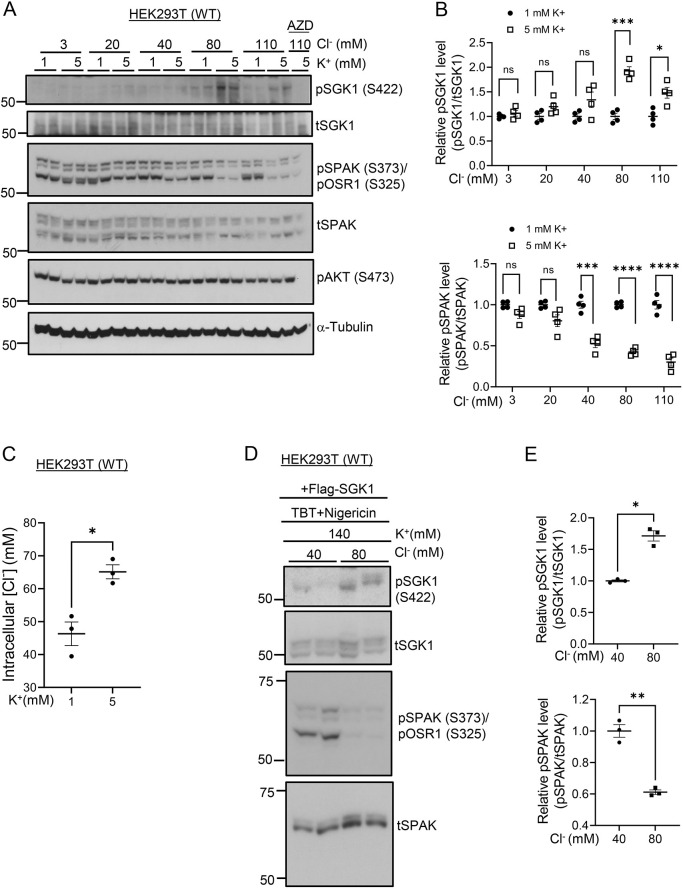
**K^+^-stimulated SGK1 phosphorylation is Cl^−^ dependent.** (A) Western blot showing the effect of extracellular K^+^ on pSGK1 (S422) under normal or low extracellular Cl^−^ conditions in WT HEK-293 cells. SPAK phosphorylation is also shown. Cells were transfected with Flag–SGK1 and subsequently serum starved overnight. Cells were then shifted to media with different Cl^−^ concentrations as described in the Materials and Methods with media K^+^ concentration maintained at 1 or 5 mM and incubated for 1 h before processing for western blotting. pSGK1 protein was detected using anti-phospho-SGK1 S422 (sc-16745, Santa Cruz Biotechnology) antibody. AZD, AZD8055 treatment. t, total. Blot shown representative of *n*=4 biological replicates. (B) Quantification of western blots from A demonstrating SGK1 (upper panel) and SPAK (lower panel) phosphorylation. All values are means±s.e.m. from four independent experiments. (C) Measurement of intracellular [Cl^−^] in HEK cells in 1 versus 5 mM extracellular [K^+^] medium. All values are means±s.e.m. from *n*=3 biological replicates. See Materials and Methods for details. (D) Western blot showing the effect of changing intracellular [Cl^–^] from 40–80 mM on pSGK1 (S422) in WT HEK-293 cells. Cells were transfected with Flag–SGK1 and subsequently serum starved overnight. Cells were then shifted to a medium with a Cl^−^ concentration of either 40 or 80 mM containing ionophores, TBT (10 µM) and nigericin (5 µM) and incubated for 1 h before processing for western blotting as described in Materials and Methods. Results are representatives of *n*=3 biological replicates. (E) Bands in the western blots from D were quantified demonstrating SGK1 and SPAK phosphorylation. All values are means±s.e.m. from 3 independent experiments. *P<0.05; **P<0.01; ***P<0.001, ****P<0.0001; ns, not significant [multiple unpaired two-tailed *t*-test (B), unpaired two-tailed *t*-test (C,E)].

Next, we addressed the electrolyte determinants of the K^+^ effect on the WNK1–mTORC2–SGK1 signaling module. Cl^−^ has been shown to bind WNK1 *in vitro* and strongly inhibit its kinase activity toward SPAK (Piala et al., 2014). Prior work further also supports the idea that, in the DCT, this WNK inhibitory effect of Cl^−^ plays a key role in K^+^ inhibition of NCC (Terker et al., 2015). Based on these observations, we speculated that Cl^−^ might stimulate WNK1–mTORC2-dependent SGK1 phosphorylation, which would provide a parsimonious mechanism for integrating K^+^-dependent NCC inhibition and ENaC stimulation. To test this hypothesis, we first examined the Cl^−^-dependence of the K^+^ effect by altering the medium [Cl^−^] from 3 mM to 110 mM. SGK1 HM phosphorylation (Fig. 6A, pSGK1, Fig. 6B, upper panel; Fig. S5) was negligible when cells were incubated in low [Cl^–^] medium ([Cl^–^]<40 mM), and was not stimulated by raising [K^+^]. At 40 mM [Cl^–^], pSGK1 was modestly stimulated, and with [Cl^–^] ≥80 mM, K^+^-stimulated SGK1 phosphorylation was markedly increased. In keeping with previous reports (Terker et al., 2015), pSPAK was high and not K^+^-inhibited in low Cl^–^, but became K^+^-inhibitable at 40 mM [Cl^–^]. The K^+^ sensitivity further increased at 80 and 110 mM [Cl^–^] (physiological concentrations). Interestingly, Cl^–^ inhibitory effect on SPAK phosphorylation (Fig. 6A, pSPAK, pOSR1; Fig. 6B, lower panel) paralleled the stimulation of pSGK1. Also of note, there was no effect of [K^+^] or [Cl^–^] on Akt phosphorylation (Fig. 6A, pAKT).

We next demonstrated that increased extracellular [K^+^] does indeed induce an increase in intracellular [Cl^–^]. We compared the intracellular Cl^−^ concentration in HEK-293 cells incubated with either 1 mM or 5 mM medium [K^+^] using a Cl^–^-sensitive fluorescent protein, mCl-YFP (Zhong et al., 2014). The cells were transfected with Cl-YFP plasmid and fluorescence signals were measured and analyzed in cells incubated with 1 and 5 mM K^+^ medium, as previously described (Kim et al., 2015; Zhong et al., 2014). Based on ionophore-clamped standard curves, the Cl^–^ concentration in HEK-293 cells treated with 5 mM K^+^ medium was measured as 65.1 mM, whereas in the presence of 1 mM extracellular K^+^ the intracellular [Cl^–^] was 46.3 mM (Fig. 6C). We next examined whether altering intracellular [Cl^–^] while maintaining [K^+^] constant would have a similar effect on pSGK1. We used the ionophores tributyltin and nigericin to clamp intracellular [K^+^] at 140 mM (a typical intracellular concentration) while clamping [Cl^–^] at either 40 or 80 mM (spanning the intracellular [Cl^–^] induced by low and high extracellular [K^+^], respectively). SGK1 HM phosphorylation was greater in the presence of high [Cl^–^] as compared to low [Cl^–^] (Fig. 6D, pSGK1; Fig. 6E, upper panel) conditions. Consistent with *in vitro* studies (Piala et al., 2014; Terker et al., 2016), WNK-dependent SPAK phosphorylation was inhibited by high [Cl^−^] (Fig. 6D, pSPAK, pOSR1; Fig. 6E, lower panel). Similar effects were observed when a wider range of Cl^−^ concentrations were employed in WNK1^−/−^, SIN1^−/−^ double knockout (KO) HEK-293 cells which had been transfected with WT WNK1 and SIN1 (Fig. S6A,B). Together, these data support the notion that intracellular [Cl^−^] stimulates mTORC2-dependent SGK1 phosphorylation. Of further note, in WNK1^−/−^ cells, pSGK1 was low and insensitive to changes in extracellular [K^+^] and [Cl^−^] (Fig. S7). Thus, we conclude that extracellular K^+^ induces Cl^–^ entry into cells, which activates mTORC2 kinase activity selectively toward SGK1 in a WNK1-dependent fashion.

### WNK1 acts as a K^+^-stimulated scaffold to promote the association of mTORC2 and SGK1, and enhance SGK1 phosphorylation

**Fig. 7.**
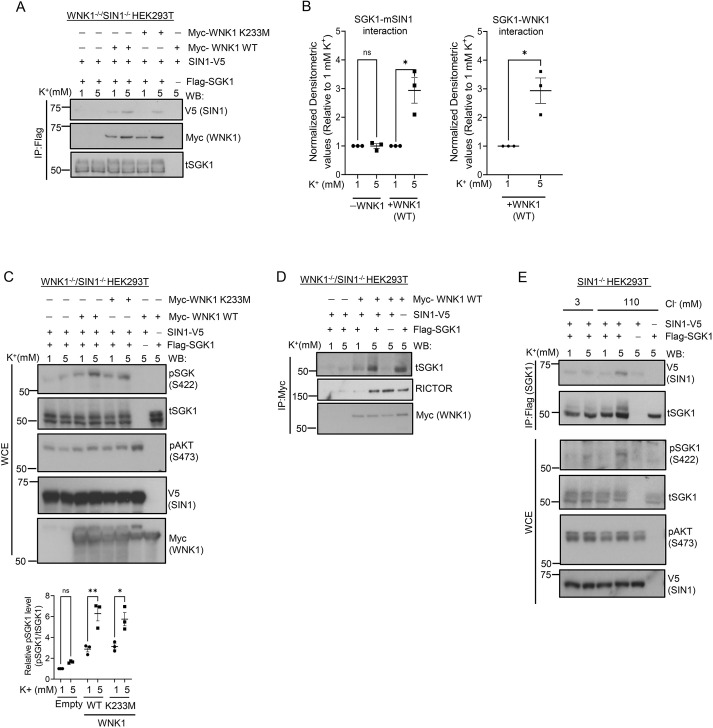
**WNK1 promotes interaction between mTORC2 and SGK1 to enhance SGK1 phosphorylation in a Cl^−^-dependent K^+^-stimulated manner.** (A) Western blot analysis of Flag immunoprecipitates derived from Flag–SGK1, Myc–WNK1 (WT and kinase-dead mutant, K233M) and SIN1–V5-transfected WNK1^−/−^ SIN1^−/−^ (double knockout) HEK-293 cells. Cells were transfected and serum starved overnight, then preincubated in 1 mM [K^+^], after which [K^+^] was shifted to 5 mM for 1 h prior to processing for IP with anti-Flag antibody and western blotted with antibodies as shown. Results are representatives of *n*=3 biological replicates. (B) Quantification of western blots from A demonstrating SGK1–SIN1 (left panel) and SGK1–WNK1 (right panel) interactions. All values are means±s.e.m. from *n*=3 biological replicates. (C) Western blot analysis of whole-cell extracts (WCEs) derived from cells in A. Upper panel, western blots were stained with antibodies as shown. pSGK1 was detected using anti-phospho-SGK1 S422 (SAB4503834, Sigma), antibody. Lower panel, quantification of bands in western blots. Note selective effect of 5 mM K^+^ on pSGK1 but not pAkt. All values are means±s.e.m. from *n*=3 biological replicates. t, total. (D) Western blot analysis of Myc–WNK1 IPs from whole-cell lysates as in A. Lysates were immunoprecipitated with anti-Myc antibody and stained for Myc, Rictor and SGK1. Note 5 mM K^+^ stimulation of Myc–WNK1 co-IP of both SGK1 (top row) and Rictor (middle row). Results are representatives of *n*=2 biological replicates. (E) K^+^ stimulated mTORC2-SGK1 interaction is Cl^−^ dependent. Western blot analysis for V5 (SIN1) of Flag (SGK1) immunoprecipitates showing the effect of extracellular K^+^ on SIN1 and SGK1 interaction under normal or low extracellular Cl^−^ conditions in Flag–SGK1, and SIN1–V5-transfected WNK1^+/+^ SIN1^−/−^ HEK-293 cells. Results demonstrate that extracellular K^+^-stimulated interaction between SGK1 and mTORC2 requires Cl^−^ (compare with Fig. 6A). In D and E, results are representatives of *n*=3 biological replicates. *P≤0.05; **P≤0.01; ns, not significant [unpaired two-tailed *t*-test (B); two-way ANOVA with Bonferroni's multiple-comparison test. (C)].

**Fig. 8.**
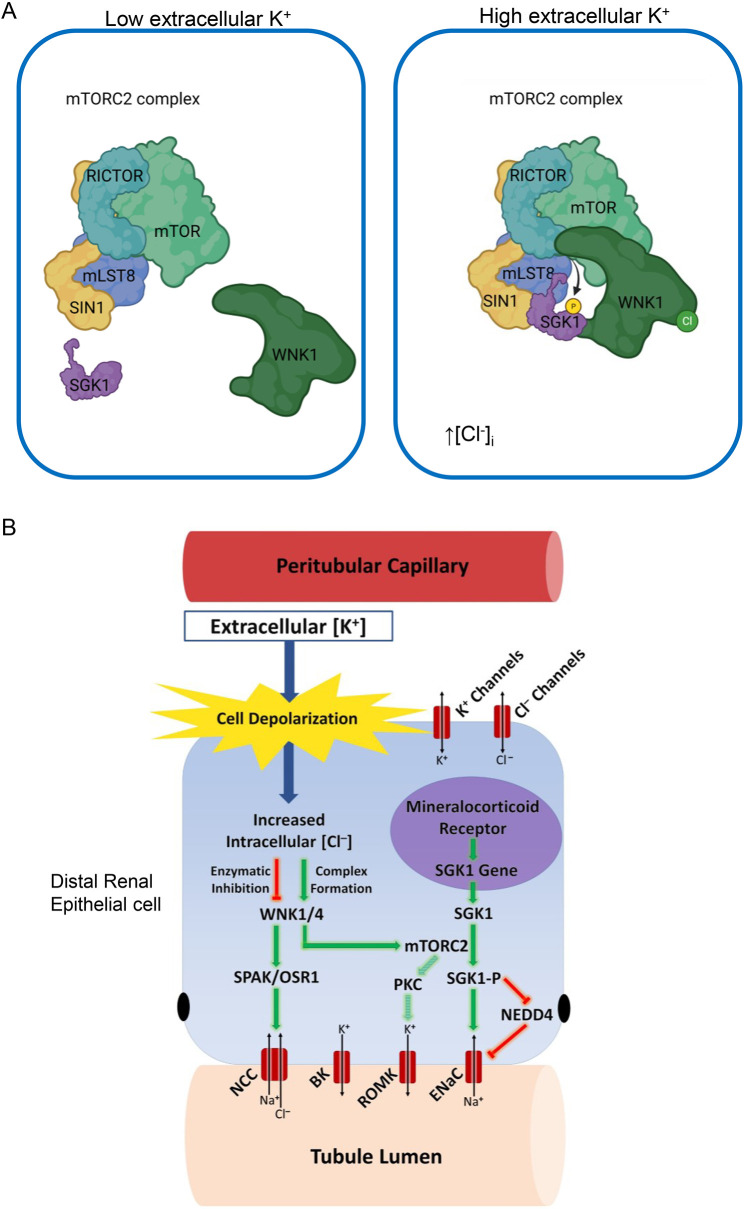
**Proposed role of WNK1 in controlling mTORC2-dependent stimulation of K^+^ secretion.** (A) WNK1 acts as a scaffold to recruit SGK1 to mTORC2 and enhance K^+^-stimulated SGK1 HM phosphorylation. Note proposed central role of Cl^−^ binding to WNK1 in mediating the effects of extracellular K^+^. According to this view, Cl^−^ triggers a conformational change in WNK1 that inhibits its kinase activity while stimulating its interaction with both SGK1 and mTORC2. (B) Schematic shows proposed interconnected events controlling apical membrane K^+^ transport in a K^+^-secreting renal epithelial cell (e.g. principal cell of the distal nephron). An increase in extracellular [K^+^] leads to depolarization of basolateral membranes, leading to Cl^−^ entry and binding to WNK4 and possibly WNK1. Proposed mechanism invokes an effect of Cl^−^ binding to alter WNK conformation to favor scaffolding (which promotes mTORC2–SGK1 interaction and SGK1 phosphorylation) while inhibiting WNK kinase activity. Activated SGK1 rapidly stimulates ENaC to increase the driving force for K^+^ secretion.

Our data establish that WNK1 enhances mTORC2-dependent phosphorylation of SGK1, and increases ENaC activity without activating Akt or PKC. This effect is markedly and selectively stimulated by K^+^ and, in contrast to SPAK–NCC regulation, WNK1 kinase activity is not implicated. Furthermore, our data provide strong support for the idea that the effect of K^+^ is mediated by Cl^–^. Particularly in light of prior publications suggesting that WNK1 can stimulate SGK1 activity through a non-catalytic mechanism (Heise et al., 2010; Xu et al., 2005a,b), these findings suggest that WNK1 might serve as a K^+^-stimulated scaffold to enhance mTORC2 interaction with SGK1 thereby promoting phosphorylation. To examine this possibility, we used WNK1 and SIN1 double KO HEK-293T cells and performed a series of co-immunoprecipitation (co-IP) experiments with selective add-back of WNK1 and SIN1 (Fig. 7; Fig. S8). First, we co-transfected the cells with Flag–SGK1, Myc–WNK1 (either WT or a kinase-dead mutant) and SIN1–V5 (Fig. 7A). The cells were adapted to 1 mM [K^+^], and then were shifted to either 5 mM or maintained in 1 mM [K^+^] for 1 h. Lysates were prepared and Flag–SGK1 was immunoprecipitated (Fig. 7A; Fig. S8A). In the absence of WNK1, SIN1 was slightly detectable by co-IP, and unaffected by [K^+^] (Fig. 7A, lanes 1 and 2). In contrast, in the presence of either WT (Fig. 7A, lanes 3 and 4) or kinase-dead WNK1 (Fig. 7A, lanes 5 and 6), SIN1–V5 was detectable in 1 mM [K^+^] and the co-IP signal was further enhanced in 5 mM [K^+^] (Fig. 7A,B). SGK1–WNK1 interaction was also stimulated by K^+^ (Fig. 7A, Myc; Fig. 7B). SGK1 phosphorylation was concomitantly increased (Fig. 7C). In further experiments, Myc–WNK1 was immunoprecipitated, and blots were stained for either SGK1 or Rictor (Fig. 7D; Fig. S8B). Notably, there was a significant WNK1–SGK1 interaction in the absence of SIN1, which was further enhanced by SIN1. Similarly, there was a strong effect of K^+^ on WNK1 co-IP of Rictor (Fig. 7D, compare lanes 3 and 4); however, SIN1 had no effect (Fig. 7D, compare lanes 4 and 6). A similar effect of WNK1 on promoting the SGK1–mTORC2 interaction was seen when SIN1 was targeted for IP (Fig. S8C). Together, these findings suggest that WNK1 directly interacts with both Rictor and SGK1. Together with previous publications demonstrating that SGK1 interacts with mTORC2 through SIN1 (Cameron et al., 2011; Lu et al., 2011), these data support a model in which WNK1 serves as a K^+^-stimulated scaffold to enhance recruitment of SGK1 to mTORC2 and hence increase its phosphorylation. Furthermore, consistent with the observation that SGK1 phosphorylation is facilitated by high intracellular [Cl^−^] (Fig. 6D), the SGK1–SIN1 interaction was markedly increased by raising extracellular [K^+^] when cells were incubated in medium with a physiological [Cl^–^], and remained negligible and unresponsive to increased extracellular [K^+^] when cells were incubated in low [Cl^–^] medium (Fig. 7E). These data support the model shown in Fig. 8A and suggest that intracellular [Cl^–^] stimulates WNK1 interaction with both SGK1 and mTORC2, and hence enhances the recruitment of SGK1 to mTORC2 and facilitates its phosphorylation. Finally, these data support a Cl^–^- and WNK1-dependent mechanism for shifting Na^+^ transport from being electroneutral (via NCC) to electrogenic (via ENaC) thereby synergistically enhancing K^+^ secretion (Fig. 8B).

## DISCUSSION

Here, we set out to investigate the signaling mechanism underlying local regulation of K^+^ secretion in kidney tubule cells. We showed previously that increased basolateral [K^+^] stimulates mTORC2-dependent SGK1 phosphorylation and ENaC activation in renal principal cells to depolarize the apical membrane and increase luminal K^+^ secretion, thereby establishing a novel homeostatic mechanism (Sørensen et al., 2019). We also showed that WNK1 markedly enhances mTORC2-dependent SGK1 phosphorylation; however, the molecular basis and functional implications of this effect remained unknown. As a first step toward addressing these questions, we generated (using CRISPR/Cas9) and characterized WNK1-deficient mpkCCD renal principal cells. These cells demonstrated markedly reduced K^+^-stimulated mTORC2-dependent SGK1 phosphorylation and ENaC activation (Fig. 2), which phenocopied mTORC2-deficient cells (Fig. 1). However, in marked contrast to mTORC2-deficient cells, Akt phosphorylation was unaffected in WNK1^−/−^ cells. Interestingly, mTORC2-dependent SGK1 activation was restored by transfection of either WT or kinase-dead WNK1 (Figs 3 and 4). That this kinase-independent activity of WNK1 is physiologically relevant was further supported by the observation that the WNK kinase inhibitor WNK463 had no effect on ENaC activity or SGK1 phosphorylation in WT mpkCCD cells at concentrations that inhibited OSR1 and SPAK phosphorylation to baseline (Fig. 5). Thus, the effects of K^+^ on both SGK1 and SPAK phosphorylation are WNK dependent; however, the mechanisms are distinct and the effects of K^+^ are opposite.

With these observations in mind, and in light of prior reports showing that extracellular K^+^ increases intracellular [Cl^–^], and that Cl^–^ binds WNK1 and inhibits its kinase activity (Piala et al., 2014; Terker et al., 2015), we explored the possibility that Cl^–^ might oppositely regulate WNK1 effects on mTORC2 activity, specifically toward SGK1. In support of this possibility, K^+^-stimulated SGK1 phosphorylation required adequate extracellular [Cl^–^] (≥80 mM), similar to that required for K^+^ inhibition of OSR1 and SPAK phosphorylation (Fig. 6A). Furthermore, we found that an increase in extracellular [K^+^] did indeed increase intracellular [Cl^–^] (Fig. 6C) and, importantly, that increasing intracellular [Cl^–^] (using an ionophore clamp) while holding [K^+^] constant increased pSGK1, whereas it decreased pSPAK. Finally, we demonstrated that WNK1 physically interacts with both SGK1 and mTORC2 and stimulates their mutual interaction in a Cl^–^-dependent fashion (Fig. 7E), consistent with it having a role as a Cl^–^ regulated scaffold that selectively enhances SGK1 (but not Akt or PKC) phosphorylation.

Together, these data support the model for K^+^-induced SGK1 regulation shown in Fig. 8A. According to this view, increased extracellular K^+^ induces a rise in intracellular Cl^–^, which binds WNK1 and alters its conformation, enhancing its interaction with both SGK1 and mTORC2 to promote SGK1 phosphorylation. SGK1-dependent activation of ENaC increases the driving force for K^+^ transport through renal outer medullary K^+^ channels (ROMK channels) and possibly BK channels (Fig. 8B). Thus, WNK1 acts as a Cl^–^ regulated scaffold that senses extracellular K^+^ and stimulates K^+^ secretion to complete a cell-autonomous homeostatic loop. These effects do not require WNK1 kinase activity (Figs 3–5), which is in fact inhibited by rising [Cl^–^] (Fig. 6D) (Piala et al., 2014); this latter effect plays a central role in mediating effects of K^+^ to inhibit SPAK phosphorylation, and hence cation-chloride cotransporters such as NCC and NKCC1 and 2 (Murillo-de-Ozores et al., 2020; Sorensen et al., 2013; Terker et al., 2015). Together, these observations suggest that Cl^–^ induces a WNK1 conformation that favors its scaffolding function while concomitantly inhibiting its kinase activity (Fig. 8A,B). It is clear from earlier work (Piala et al., 2014) that Cl^–^ induces a change in WNK1 conformation that disrupts catalytic activity; however, how this conformation favors interaction with SGK1 and mTORC2 requires further study. Interestingly, the functional and co-IP data demonstrate that the effect is selective for SGK1, whereas Akt phosphorylation is unaffected.

Cells in the ASDN express both WNK1 and WNK4 (Shekarabi et al., 2017), and we cannot rule out a role for WNK4. We have not succeeded in making a selective WNK4^−/−^ line mpkCCD cells. It is notable that the effect of extracellular K^+^ on SGK1 phosphorylation and ENaC activity is completely abrogated in WNK1^−/−^ cells (Fig. 2), supporting its central role. However, a previous study found that mice with knock-in of a Cl^–^-binding deficient mutant of WNK4 had a defect in ENaC activation (Chen et al., 2019), and it is possible that WNK4 also contributes to this mechanism. Prior studies have shown interactions of WNK1 and WNK4, and it is possible that WNK1, independent of its kinase activity interacts with other WNKs to modulate their activity and induce SGK1 phosphorylation; also it is possible that the kinase activity of WNK4 (or conceivably another WNK, such as WNK3) is implicated. Along these lines, it is further notable that the WNK kinase inhibitor WNK-463 did, at high concentrations, inhibit mTORC2 phosphorylation of SGK1 (Fig. 5). The concentrations required were 10-fold higher than those inhibiting WNK-dependent SPAK phosphorylation, and hence this could be an off-target effect. In any case, our data strongly suggest that WNK1, independent of its kinase activity, serves as a scaffold to bring together SGK1 and mTORC2 and enhance SGK1 phosphorylation. Further studies will be needed to address the role of WNK4, including its kinase activity. It is also important to note that a shorter variant of WNK1 (KS-WNK1), which lacks kinase activity and is highly expressed in aldosterone-sensitive distal nephron (ASDN) principal cells (in an aldosterone-regulated fashion), interacts with both WNK1 and WNK4, and could play a significant role in modulating ENaC and other transporters, such as ROMK, BK and NCC channels (Argaiz et al., 2018; Cheng et al., 2013; Liu et al., 2009; Ray et al., 2021; Shekarabi et al., 2017; Wade et al., 2006). It will be of interest to explore these possibilities in further studies.

It is also interesting to consider the potential implications of this mechanism for other processes, such as thermogenesis, inflammation and immune modulation. Recent studies suggest that Cl^−^-regulated inflammatory responses might be implicated in a variety of diseases, including inflammatory bowel disease, cystic fibrosis, atherosclerosis and hypertension (Lamkanfi and Dixit, 2014; Madhur et al., 2021; Scambler et al., 2019). One recent publication implicated WNK1 in mediating Cl^−^ modulation of NLRP3 inflammasome activity (Mayes-Hopfinger et al., 2021) and a separate study similarly identified SGK1 as an important inflammasome modulator (Clauzure et al., 2021); however, neither study addressed the potential connection between SGK1 and WNK1. mTORC2 modulation has also been implicated in responses to acute cold (Allu et al., 2021; Sanchez-Gurmaches et al., 2018) – one of these reports, showed that mTORC2 plays a central role in rapid direct responses to acute cold, stimulating catabolic and inhibiting anabolic activity in the absence of sympathetic nervous system activation (Allu et al., 2021). However, the cell-autonomous sensing mechanism to control mTORC2 activity was not elucidated.

Finally, in regard to the implications of our present findings for K^+^ homeostasis, it is worth emphasizing that concomitantly inhibiting WNK1 kinase activity through K^+^ while stimulating its interactions with SGK1 and mTORC2 provides a parsimonious mechanism for rapidly shifting Na^+^ transport from electroneutral NaCl reabsorption (which defends extracellular fluid volume) to electrogenic reabsorption (which drives K^+^ secretion to bring down extracellular [K^+^]) (Pearce et al., 2022; Sørensen et al., 2019) (Fig. 8B). Modulators of this aldosterone-independent mechanism for K^+^ secretion might provide new avenues for treating hyperkalemia, particularly when induced by inhibitors of the renin–angiotensin–aldosterone system.

## MATERIALS AND METHODS

### Expression constructs

Flag–SGK1 (WT), SGK1 (K127M) and SIN1–V5, were generated as described previously (Lu et al., 2011). Myc-tagged WT WNK1 (1–491) and Myc–WNK1 (1-491) (K233M) expression constructs were generous gifts from Drs Melanie H. Cobb (UT Southwestern, Dallas, TX, USA) and Chou-long Huang (University of IOWA, IOWA City, IA, USA; Xu et al., 2005b). Myc–L-WNK1 (WT) and HA–L-WNK1 (WT and K233M) expression constructs were generous gifts from Dr David H. Ellison (Oregon Health and Science University, Portland, OR, USA), and Drs Thomas R. Kleyman and Marcelo D. Carattino, University of Pittsburgh, PA, USA, respectively.

### Cell culture, transfection and treatment

mpkCCDc14 (mpkCCD) (a kind gift from Alain Vandewalle, Institut National de la Santé et de la Recherche Médicale), mpkCCD WNK1^−/−^ and mpkCCD RICTOR^−/−^ cells were maintained in modified DMEM/Ham's F12 (1:1) medium (‘regular medium’) as described previously (Bhalla et al., 2005). mpkCCD WNK1^−/−^ cells were transfected using Lipofectamine 3000 (Thermo Fisher Scientific).

HEK-293T (ATCC), HEK-293T WNK1^−/−^ (Roy et al., 2015), HEK-293T SIN1^−/−^ (Gleason et al., 2019) and HEK-293T WNK1^−/−^ SIN1^−/−^ (double knockout) cells were grown in DMEM (DME H-21, CCFAA005, UCSF Cell culture facility) supplemented with 10% FBS and 1% penicillin and streptomycin antibiotics. These cell lines were routinely tested for mycoplasma contamination. All cell lines were acquired from reputable sources, but have not recently been authenticated. Cells were transfected using polyethylenimine (PEI). For all experiments, cells were serum-starved overnight. For experiments with different K^+^ concentrations, DMEM without KCl was used. K^+^-free DMEM was supplemented with KCl, NaCl or choline chloride to get the desired concentrations of K^+^ in the medium as indicated in the figure legends.

For experiments with different extracellular Cl^−^ concentrations, DMEM without KCl and NaCl was used (UCSF cell culture facility). The medium was supplemented with sodium gluconate and NaCl to vary Cl^−^ concentrations as indicated in the figures. Different K^+^ concentrations were achieved by adding potassium gluconate and sodium gluconate or KCl and NaCl to the media to maintain same osmolarity in all cultures.

### Measurement of intracellular Cl^−^

HEK-293 cells were transfected with a modified Cl^−^ sensor YFP plasmid, monomeric Cl-YFP (Zhong et al., 2014) in 10 cm^2^ plates. At 24 h post transfection, cells were re-plated in 96-well plates coated with poly-L-lysine. After 24 h, cells were incubated with 1 and 5 mM [K^+^] solution (composed of 110 mM NaCl, 2 mM CaCl_2_, 1.2 mM MgCl_2_, 10 mM HEPES, 25 mM glucose, and 30 mM Na-gluconate, pH 7.2, with 1 and 5 mM [K^+^] adjusted with equimolar potassium gluconate and sodium gluconate) for 30 min–1 h prior to measurement of fluorescent signals. To produce a standard curve, cells were incubated with high K^+^ solutions composed of 140 mM K^+^ (KCl or potassium gluconate), 2 mM CaCl_2_, 1.2 mM MgCl_2_, 10 mM HEPES and 25 mM glucose, containing different chloride concentrations (adjusted with equimolar KCl and potassium gluconate), and the ionophores nigericin (5 µM; cat. #B7644, APExBIO) and tributyltin (10 µM; cat. #T50202, Sigma Aldrich). Fluorescence signals were measured on a Synergy H4 Hybrid Multi-Mode Microplate Reader with excitation at 485 nm and emission at 520 nm. Fluorescence intensity values measured in each solution were normalized to that of 2 mM [Cl^−^] standard solution in each well.

For experiments with modulation of intracellular [Cl^–^], cells were incubated for 1 h in high K^+^ solutions with different [Cl^–^] in presence of nigericin (5 µM) and tributyltin (10 µM) to clamp intracellular and extracellular [Cl^–^].

### Generation of knockout mpkCCD and HEK-293T cells by CRISPR/Cas9

For CRISPR knockouts of the *WNK1* and *RICTOR* gene in mpkCCD cells, WNK1 sgRNA CRISPR Lentiviral vector (pLenti-U6-sgRNA-PGK-Neo; #504391140195, Applied Biological Materials) or RICTOR sgRNA CRISPR Lentiviral vector (#395791140195, Applied Biological Materials) and Cas9-expressing lentiviral vector (pLenti-EF1a-Cas9-Puro; #K002, Applied Biological Materials) were used. The lentiviral plasmid DNA were packaged into lentivirus by co-transfection with Virapower (Invitrogen) in HEK-293FT cells. Supernatant containing lentivirus was used to infect mpkCCD cells and then infected cells were selected in puromycin (5 μg/ml) and neomycin (for WNK1^−/−^) or hygromycin (for RICTOR^−/−^). Single colonies were selected either by fluorescence-activated cell sorting (FACS) into a 96-well plate, or using cloning rings and expanded and tested for WNK1 and Rictor expression by western blotting.

WNK1 SIN1 double-knockout (WNK1^−/−^ SIN1^−/−^) HEK-293T cells were generated by knocking out the *SIN1* gene in HEK-293T WNK1^−/−^ cells (Roy et al., 2015) cells using the CRISPR/Cas9 Lentiviral system. SIN1 sgRNA CRISPR/Cas9 (All in one) lentiviral vector containing a sgRNA targeting the 3′ end of exon 3 of the human *MAPKAP1* genomic locus, and a Cas9 (Gleason et al., 2019) expressing locus was used for lentivirus production. HEK-293 WNK1^−/−^ cells were then infected with the lentivirus and subsequently single clones of the WNK1 SIN1 double-knockout cell lines (WNK1^−/−^ SIN1^−/−^) were generated as described above. The knockout cells were tested for SIN1 expression by western blotting.

### Measurement of ENaC-dependent Na^+^ transport

For electrophysiological measurements, mpkCCDc14 cells were seeded on type I collagen-coated filters (Transwell, pore size 0.4 µm, Corning Costar) until the cell monolayers reached a transepithelial resistance of >1000 Ω.cm^2^. They were then maintained in serum-free plain DMEM for at least 16 h before treatment with aldosterone (1 µM for 3 h) to induce endogenous SGK1 expression. For extracellular [K^+^] stimulation–recovery experiments, cells were adapted for at least 2 h to 1 mM or 3 mM [K^+^] on the basolateral side. At *t*=0, the medium [K^+^] was increased by addition of potassium gluconate or KCl or equimolar sodium gluconate or sodium chloride and incubated for 1 h prior to measurement of amiloride-sensitive current. Transepithelial resistance and potential difference across the cell monolayer were measured using Millepore ERS voltohmmeter (MilliCell ERS; Millipore) (Lu et al., 2010). The equivalent short-circuit current was calculated using Ohm's law. Amiloride (10 µM; cat. #A7410, Sigma-Aldrich) was added to the medium on the apical side at the end of the experiments to derive amiloride-sensitive component of the current. Amiloride almost completely (>98%) inhibited the total current, indicating its ENaC dependence. In some experiments, cells were adapted to 5 mM [K^+^] and then transferred to media with different [K^+^] as specified.

In inhibitor study experiments, cells were treated for 15 min with inhibitors – WNK463 (0.1–1 µM, apical and basolateral sides; cat. #A16205, Adooq Bioscience), AZD8055 (1 µM, apical and basolateral sides; cat. #S1555, SelleckChem), or equal volumes of vehicle as the control prior to raising basolateral K^+^ concentration from 1 to 5 mM.

### Patch-clamp and single and multi-channel current measurement in cells

Cells were plated on 5×5 mm glass coverslips coated with poly-D-lysine, and coverslips were transferred to the recording chamber of the microscope. For the single channel recording, an Axon200B patch-clamp amplifier was used to record the channel current. Amiloride-sensitive ENaC single channel and multichannel activities were measured at −60 mV holding potential. Pipette solution contained (in mM): 140 LiCl, 2 MgCl_2_ and 10 HEPES, pH 7.4; bath solution contained (in mM): 140 NaCl, 5 KCl, 1.8 CaCl_2_, 1.5 MgCl_2_, 10 HEPES (pH 7.4). Gap-free single-channel current data from gigaohm seals were acquired and subsequently analyzed with an Axopatch 1D (Axon Instruments) patch-clamp amplifier interfaced via a Digidata 1440A (Axon Instruments) to a computer running the pClamp 10.2 (Axon Instruments). The channel open probability (*P*_o_) was calculated from the channel number (*N*) and *NP*_o_ (a product of channel number and open probability), which was calculated from data samples of 60-s duration in the steady state as follows:

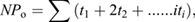
where *t*_i_ is the fractional open time spent at each of the observed current levels.

In multichannel cell attached mode net current (in pA) is measured in individual patches at specific holding potentials in cell attached mode. The measured pA signal in this case reflects the sum of all the channel activities in one patch.

### Immunoblotting

To determine protein expression levels, western blot analysis was performed as previously described (Lu et al., 2010; Sørensen et al., 2019). Briefly, cells were lysed in 1% Triton X-100 buffer (40 mM HEPES pH 7.5, 1 mM EDTA pH 8, 10 mM sodium pyrophosphate, 10 mM glycerophosphate, 50 mM sodium fluoride, 120 mM sodium chloride and 1% Triton X-100) containing complete protease inhibitor cocktail and PhoSTOP phosphatase inhibitor. Total protein (40 μg) from each cell extract supernatant was electrophoresed on 8% polyacrylamide gels. The blots were then probed with primary antibodies (Table S1) followed with horseradish peroxidase (HRP)-conjugated secondary antibodies. Each experiment was repeated more than three times. The bands corresponding to the proteins of interest were quantified on scanned films using NIH Image J software, as previously described.

### Immunoprecipitation

Cell lysates were prepared using 1% CHAPS buffer (40 mM HEPES pH 7.5, 1 mM EDTA pH 8, 10 mM sodium pyrophosphate, 10 mM glycerophosphate, 50 mM sodium fluoride, 120 mM sodium chloride and 1% CHAPS) containing complete protease inhibitor cocktail and PhoSTOP phosphatase inhibitor (cat. #04906837001, Roche), and centrifuged at 14,000 ***g*** for 10 min. Supernatant (cell extract) was removed and protein content was estimated by performing a Bradford assay. Immunoprecipitation was performed using anti-FLAG M2 affinity gel (#A2220, Sigma-Aldrich), anti-Myc agarose gel (#20168, Thermo Fisher Scientific), and anti-V5-affinity gel (#A7345, Sigma). To immunoprecipitate Flag–SGK1, SIN1–V5 and Myc–WNK1, 100–200 µg of cell extract protein was rotated overnight at 4°C with 20 µl of 50% slurry anti-FLAG or anti-V5 affinity gel or 25% slurry anti-Myc affinity gel respectively. The agarose beads were collected by centrifugation (5000 ***g*** for 1 min), washed three times with 1% CHAPS cell lysis buffer. Protein was eluted from the beads by adding Laemmli sample buffer and subsequently boiled and denatured, and then separated by SDS-PAGE and transferred to PVDF membrane as described under immunoblotting. Each experiment was repeated at least three times independently with similar results.

### Statistics

GraphPad Prism (GraphPad Software, USA) and Microsoft Excel were used for data analysis. Comparison between two groups was performed by unpaired two-tailed *t*-test. Comparison between more than two groups was analyzed by one-way ANOVA with a Bonferroni's multiple comparison post-test. Statistical test used are stated in each figure legend. *P*<0.05 is considered significant and *P*-values are either shown directly in the figures or symbols for *P*-values are explained in figure legends.

## Supplementary Material

10.1242/joces.260313_sup1 Supplementary information
