# Supplementary material for: WNK1 is a chloride-stimulated scaffold that regulates mTORC2 activity and ion transport
Source: J Cell Sci. 2022 Dec 5;135(23):jcs260313. doi: 10.1242/jcs.260313 (PMC9789407; doi:10.1242/jcs.260313)
Supplement: Supplementary information [file joces-135-260313-s1.pdf]

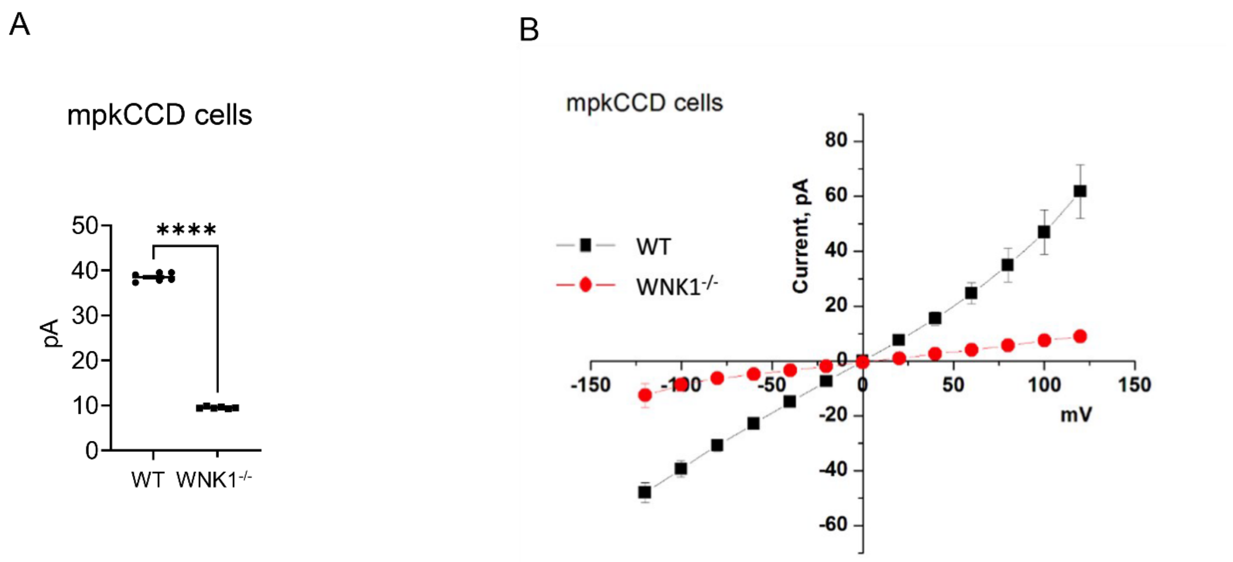

Fig. S1. WNK1 is required for K^+-induced ENaC activity. (A) ENaC current measured in the cell-attached (multi-channel) configuration in WT vs. WNK1$^{-/-}$ (clone 10) mpkCCD cells at steady-state level. ENaC current was assessed as amiloride-sensitive current at a clamp potential of –60 mV. The bath [K^+] was maintained at 5 mM. All values are means ± SEM; ****P<0.0001 by unpaired t-test. pA, picoamperes. (B) Current-voltage relationship (I-V) of voltage-clamped ENaC in WT and WNK1$^{-/-}$ mpkCCD cells. ENaC current was measured in the cell-attached (multi-channel) configuration in WT (black sequres) vs. WNK1$^{-/-}$ (red circles) mpkCCD cells at steady-state level. Cells were maintained in the media containing 5 mM [K+]. ENaC current was assessed as amiloride sensitive current at a clamp potential of –60 mV. A and B, n=6 in each group.

Journal of Cell Science • Supplementary information

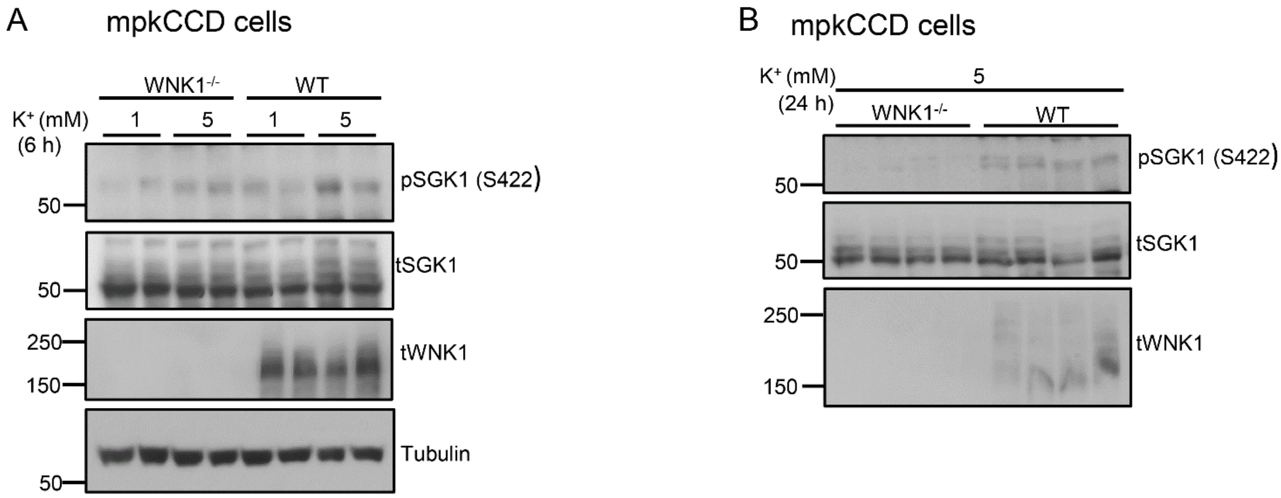

Fig. S2. WNK1 is required for K^+-stimulated SGK1 phosphorylation. (A & B) Representative western blots showing the effect of extracellular K^+ on SGK1 phosphorylation in WT and WNK1$^{-/-}$ mpkCCD cells over a course of 6 h (A) and 24 h (B). WT and KO cells were grown on Transwell filters, adapted to 1 mM [K^+] on the basolateral side, and then the medium [K^+] was raised to 5 mM and kept for either 6 hour (A) or 24 h (B) before harvesting the cells and subsequent western blot analysis for various proteins as indicated.). pSGK1 and WNK1 proteins were detected using anti–phospho-SGK1 S422 (sc-16745, Santa Cruz Biotechnology) and anti–WNK1 (AF2849, R&D Systems) antibodies, respectively. Note that stimulatory effects of 5 mM [K^+] on pSGK1 are observed in WT cells and the effect is maintained over a long period of time. The stimulatory effect of extracellular K+ on pSGK1 was not observed in WNK1-deleted cells. A and B, results are representatives of n=3 biological replicates.

Journal of Cell Science • Supplementary information

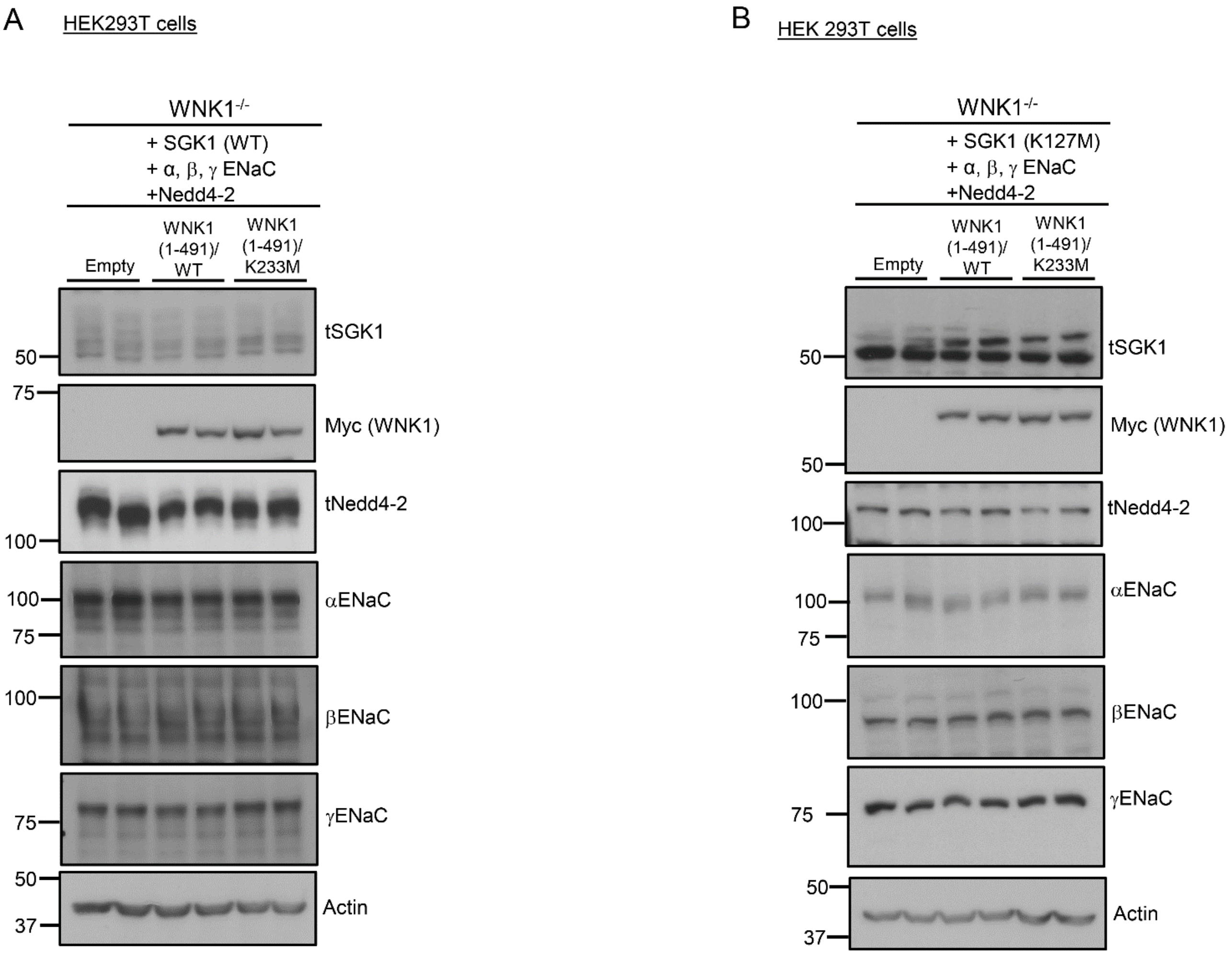

Fig. S3. Western blot analysis to determine the expression levels of different proteins in transfected HEK 293 cells. (A and B) WNK1$^{-/-}$ HEK 293 cells were transfected with β ,α, and γENaC, Nedd4-2, WT (aa 1-491) or kinase-dead (aa 1-491) (K233M) WNK1 and with either WT-SGK1 (A) or kinase-dead SGK1 (K127M) (B). 48 h post transfection cells were analysed for the protein expressions (as indicated) prior to patch-clamp experiemnts. A and B, results are representatives of n=3 biological replicates.

Journal of Cell Science • Supplementary information

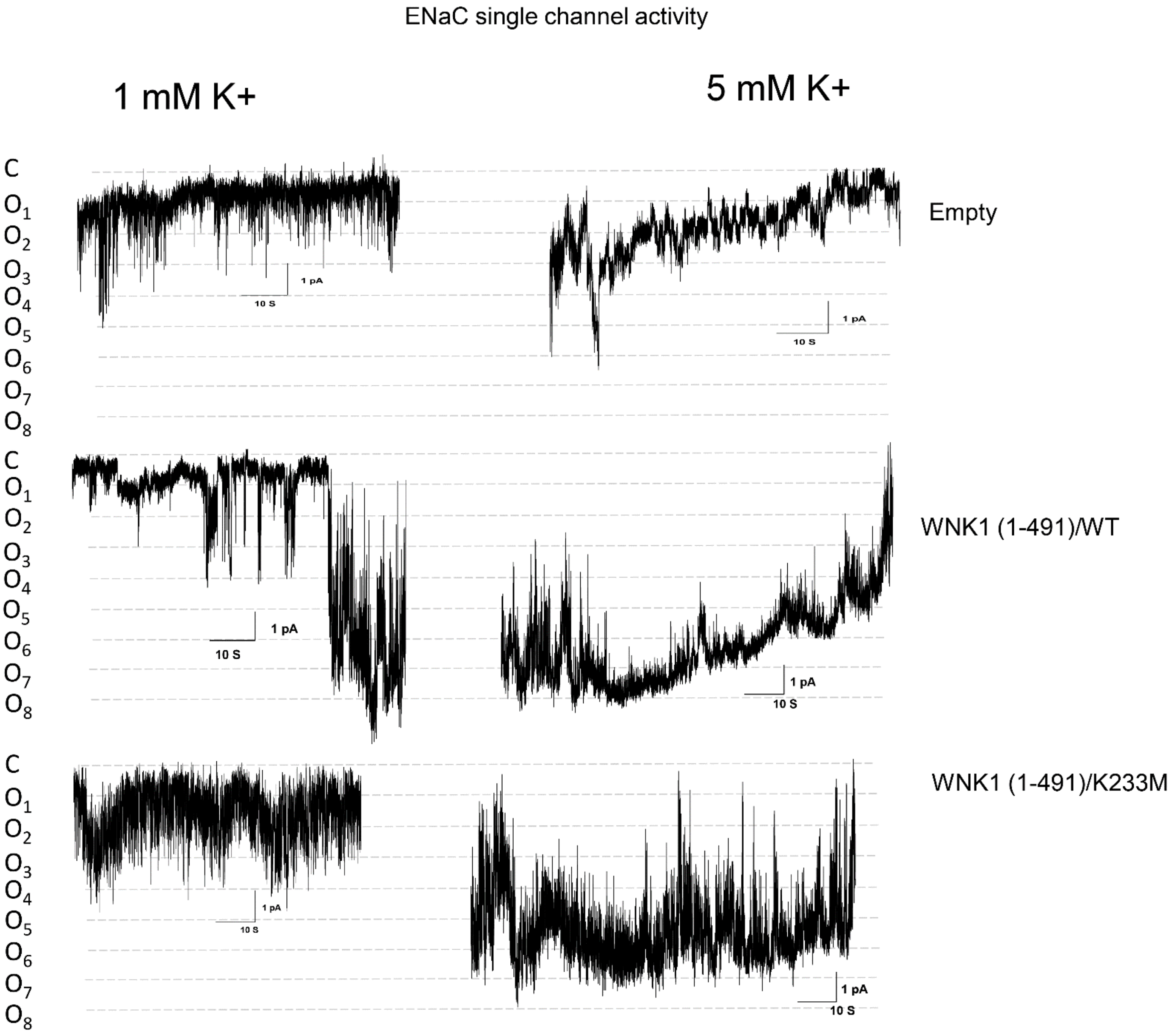

Fig. S4. Representative current traces of ENaC channels recorded in the transfected WNK1-/- HEK293 cells 1 h post incubation in 1 or 5 mM [K+]. Cells from Fig. S3, were adapted to 1 mM [K^+], and then bath [K^+] was either raised to 5 mM or kept at 1 mM for 1 hour prior to measurement of amiloride-sensitive current by patch-clamp.See Methods for experimental details. Results are representatives of n=5 in each group.

Journal of Cell Science • Supplementary information

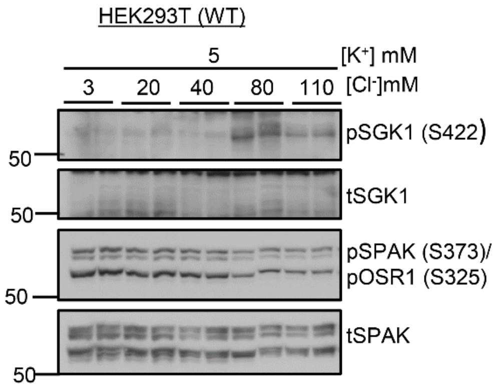

Fig. S5. K^+ stimulated WNK1/mTORC2-dependent SGK1 phosphorylation is Cl^- dependent. (A) Western blot for pSGK1 under low or normal extracellular Cl^- conditions in WT HEK293 cells. SPAK phosphorylation is also shown. Cells were transfected with Flag-SGK1 and subsequently serum starved overnight. Cells were then shifted to media with different Cl^- concentrations as described in 'Methods' with media K^+ concentration maintained at 1 or 5 mM as indicated and incubated for 1 h before processing for western blot. Results are representatives of n=4 biological replicates.

Journal of Cell Science • Supplementary information

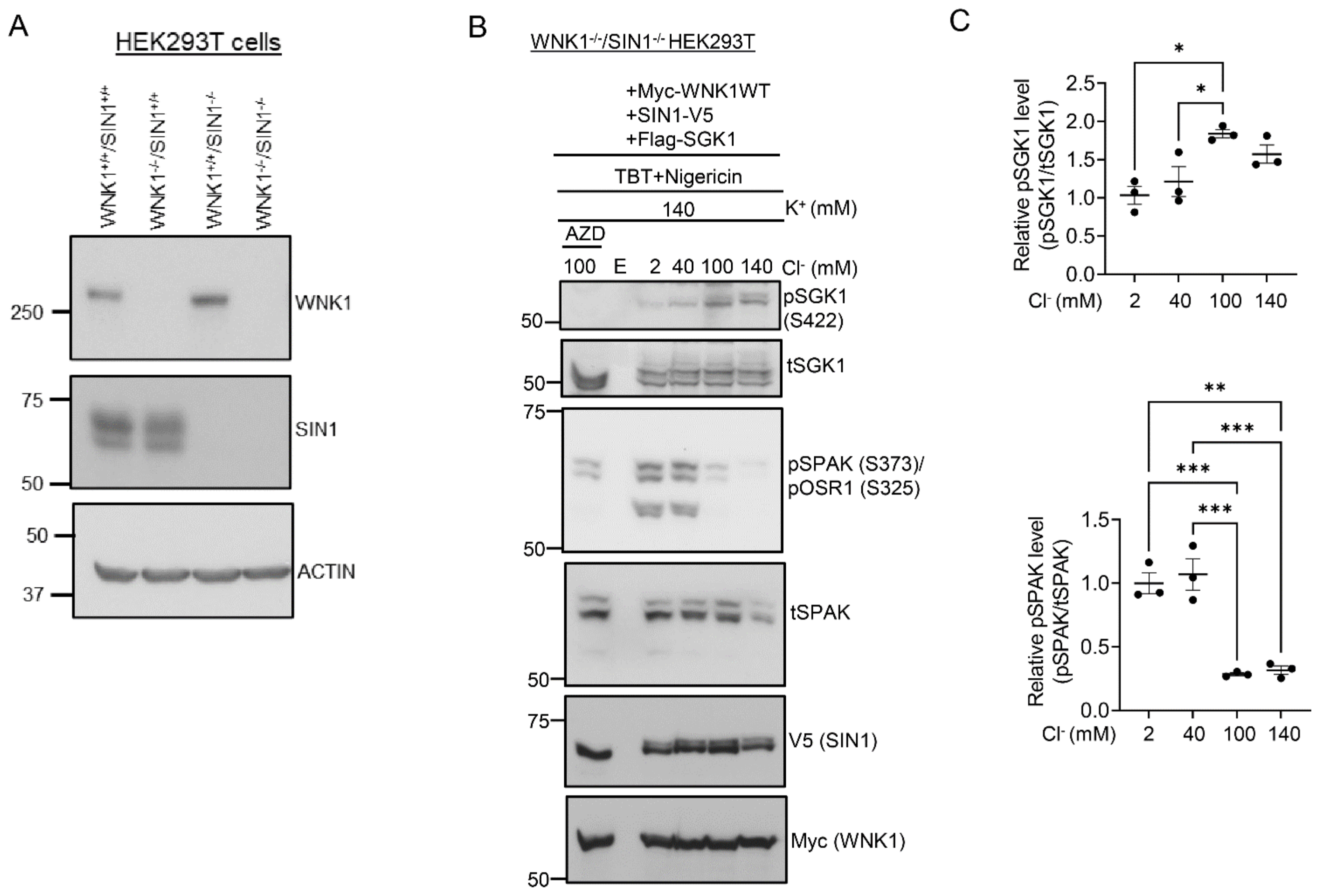

Fig. S6. (A) Generation and validation of WNK1 and SIN1 knockout HEK293 cell lines. Western blot analyses of WNK1 and SIN1 proteins in WT, and WNK1, SIN1 and WNK1/SIN1 double KO HEK293T clones generated using CRISPR/Cas9 (see Methods for details). WNK1 protein was detected using anti–WNK1 (4979, CST) antibody. Results are representatives of n=2 biological replicates. (B) Western blot showing the effect of intracellular [Cl^-] changes on pSGK1 (S422) in WNK1$^{-/-}$/SIN1$^{-/-}$ HEK-293 cells transfected with WT Myc-WNK1, SIN1–V5 and Flag-SGK1. Cells were serum starved overnight, shifted to media with different Cl^- concentrations (2, 40, 100 and 140 mM) containing ionophores, TBT (10 μM) and nigericin (5 μM) to clamp intracellular [K^+] and [Cl^-], and incubated for 1 h before processing for western blot as described in 'Methods'. Western blots were stained with antibodies as indicated. (C) Bands in the WBs from the (B) were quantitated demonstrating SGK1 and SPAK phosphorylation. All values are means ± SEM; * $P \leq 0.05$, **$P \leq 0.01$, ***$P \leq 0.001$ by one-way ANOVA with Bonferroni's multiple-comparison test. Results are representatives of n=3 biological replicates. E, Empty lane.

Journal of Cell Science • Supplementary information

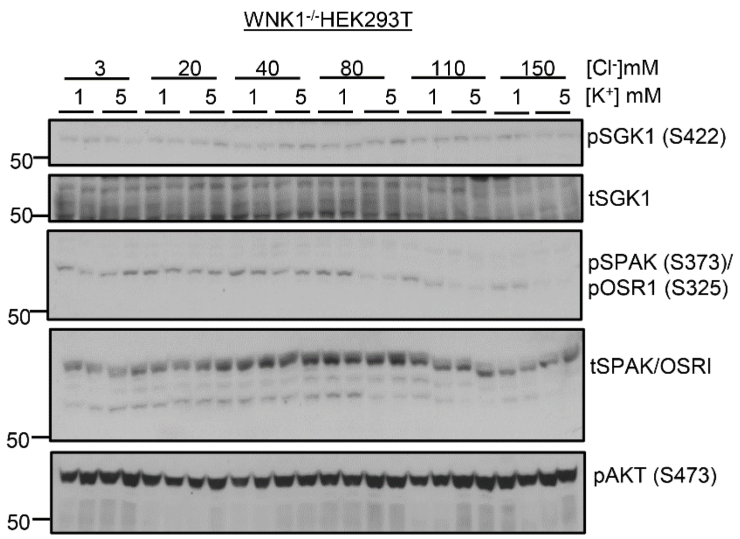

Fig. S7. Western blot for pSGK1 under low or normal extracellular Cl^- conditions in WNK1$^{-/-}$ HEK 293T cells. SPAK phosphorylation is also shown. Cells were transfected with Flag-SGK1 and subsequently serum starved overnight. Cells were then shifted to media with different Cl^- concentrations as described in 'Methods', with media K^+ concentration maintained at 1 or 5 mM as indicated and incubated for 1 h before processing for western blot. Results are representatives of n=3 biological replicates.

Journal of Cell Science • Supplementary information

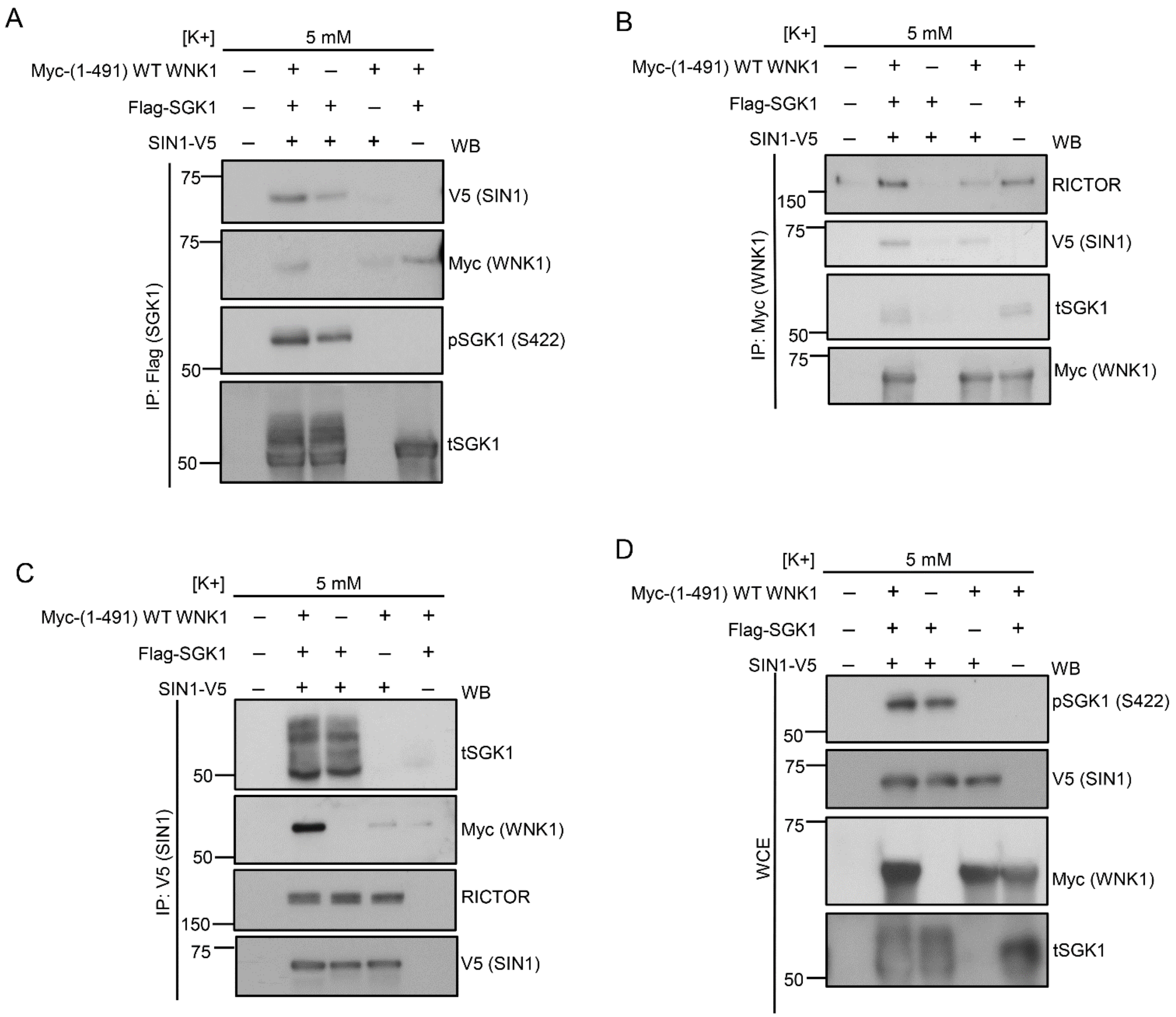

Fig. S8. WNK1, SGK1 and mTORC2 form a complex. (A-C) Western blot (WB) analysis of Flag- (A), Myc- (B) and V5- (C) immunoprecipitates (IPs) derived from Flag–SGK1, Myc-WNK1 (WT) and SIN1–V5-transfected WNK1$^{-/-}$/SIN1$^{-/-}$ HEK-293T cells. Cells were transfected and harvested 48 h post-transfection in regular DMEM with 5 mM extracellular K^+. (D) Western blot analysis of whole-cell extracts (WCEs) derived from the experiments in (A-C). Note that, WNK1 and mTORC2 component mSIN1, and Rictor, coimmunoprecipitate with SGK1 which indicate that mTORC2, SGK1 and WNK1 exist in the same complex. The amount of mSIN1 interacting with SGK1 and SGK1 HM phosphorylation were increased in presence of WNK1 (A, first and third panels). WNK1 can physically interact with SGK1 in absence of a stable mTORC2 complex (A, second panel; B, third panel). A and D, results are representatives of n=3 biological replicates. B and C, results are representatives of n=2 biological replicates.

Journal of Cell Science • Supplementary information

Table S1. Primary antibodies dilution, source and validation reference. Abbrev: CST, Cell Signaling Technology; SCBT, Santa Cruz Biotechnology, Inc.

Antibody	Host	Dilution	Source	Reference
pSGK1 (S422)	Rabbit	1000	sc-16745, SCBT	
pSGK1 (S422)	Rabbit	1000	SAB4503834, Sigma	
tSGK1	Rabbit	2000	5188, Sigma	
pAKT (S473)	Rabbit	2000	4060, CST	
tAKT	Rabbit	2000	9272, CST	
c-Myc	Rabbit	2000	C3956, Sigma	
V5-HRP	Mouse	1000	R961-25, ThermoFishher Scientific	
HA-HRP	Rabbit	1000	AB128131, Abcam	
SIN1	Mouse	1000	05-1044 Millipore	
Cas9	Mouse	1000	Y300079, abm	
Tubulin	Mouse	5000	T9026, Sigma	
α-ENaC	Rabbit	1000	Loffing	Sorensen et al., 2013
β-ENaC	Rabbit	1000	Loffing	Wagner et al., 2008
γ-ENaC	Rabbit	1000	SPC-405, StressMarq	
β-Actin	Mouse	5000	A2228, Sigma	
Rictor	Rabbit	1000	2114, CST	
Rictor	Goat	1000	50678, SCBT	
pNedd4-2 (S328)	Rabbit	1000	ab95399, Abcam	
tNedd4-2	Rabbit	1000	ab46521, Abcam	
WNK1	Goat	1000	AF2849, R&D Systems	
WNK1	Rabbit	1000	4979, CST	
WNK1	Rabbit	1000	NBP2-75712, Novus Biologicals	
pSPAK (S373)/pOSRI (S325)	Rabbit	1000	07-2273, Millepore	
tSPAK/OSR1	Rabbit	1000	07-2271, Millepore	

SUPPLEMENTAL REFERENCES

Sorensen, M. V., Grossmann, S., Roesinger, M., Gresko, N., Todkar, A. P., Barmettler, G., Ziegler, U., Odermatt, A., Loffing-Cueni, D. and Loffing, J. (2013). Rapid dephosphorylation of the renal sodium chloride cotransporter in response to oral potassium intake in mice. *Kidney Int* **83**, 811-24.

Wagner, C. A., Loffing-Cueni, D., Yan, Q., Schulz, N., Fakitsas, P., Carrel, M., et al. (2008). Mouse model of type II Bartter's syndrome. II. Altered expression of renal sodium- and water-transporting proteins. *Am J Physiol Renal Physiol* 294, F1373-1380.
