# Supplementary material for: WNK1 is a chloride-stimulated scaffold that regulates mTORC2 activity and ion transport
Source: J Cell Sci. 2022 Dec 5;135(23):jcs260313. doi: 10.1242/jcs.260313 (PMC9789407; doi:10.1242/jcs.260313)
Supplement: Supplementary file 1 [file joces-135-260313_review_history.pdf]

Journal of Cell Science

WNK1 is a chloride-stimulated scaffold that regulates mTORC2-activity and ion transport

Bidisha Saha, Deise C. A. Leite-Dellova, John Demko, Mads Vaarby Sørensen, Enzo Takagi, Catherine Gleason, Waheed Shabbir and David Pearce
DOI: 10.1242/jcs.260313

Editor: John Heath

Review timeline

Original submission:	8 June 2022
Editorial decision:	3 August 2022
First revision received:	30 September 2022
Accepted:	26 October 2022

Original submission

First decision letter

MS ID#: JOCES/2022/260313

MS TITLE: WNK1 is a chloride-dependent scaffold that regulates mTORC2-activity and ion transport

AUTHORS: Bidisha Saha, Deise Carla Almeida Leite Dellova, John Demko, Mads Vaarby Sorensen, Enzo Takagi, Catherine Gleason, Waheed Shabbir, and David Pearce
ARTICLE TYPE: Research Article

We have now reached a decision on the above manuscript.

To see the reviewers' reports and a copy of this decision letter, please go to: https://submit-jcs.biologists.org and click on the 'Manuscripts with Decisions' queue in the Author Area. (Corresponding author only has access to reviews.)

As you will see, the reviewers raise a number of criticisms that prevent me from accepting the paper at this stage. They suggest, however, that a revised version might prove acceptable, if you can address their concerns. If you think that you can deal satisfactorily with the criticisms on revision, I would be pleased to see a revised manuscript. We would then return it to the reviewers.

Please ensure that you clearly highlight all changes made in the revised manuscript. Please avoid using 'Tracked changes' in Word files as these are lost in PDF conversion.

I should be grateful if you would also provide a point-by-point response detailing how you have dealt with the points raised by the reviewers in the 'Response to Reviewers' box. Please attend to all of the reviewers' comments. If you do not agree with any of their criticisms or suggestions please explain clearly why this is so.

Reviewer 1

Advance summary and potential significance to field

WNK1 mTORC2 Journal of Chemical Sciences

© 2022. Published by The Company of Biologists under the terms of the Creative Commons Attribution License (https://creativecommons.org/licenses/by/4.0/).

The authors of this study investigate the role that the WNK1 kinase may play in controlling the ability of the mTORC2 complex in regulate the phosphorylation and activation of the SGK1 protein kinase. The authors' data suggest that in response to high extracellular K+ and high [Cl-]i that WNK1 and SGK1 are recruited to the mTORC2 complex and that this is required for optimal phosphorylation of SGK1 at its hydrophobic motif. Interestingly the authors' data suggest that the ability of WNK1 to promote mTORC2 mediated activation of SGK1 is not dependent upon its kinase activity. The data are fairly convincing, and the study would make a useful contribution to the literature in this area and potentially stimulate further research.

Comments for the author

Below I list some straightforward points that the authors should consider when revising in their study:

1. In fig.3 the authors only study the effect of 5mM K+ at a single timepoint. To demonstrate that SGK1 phosphorylation is constantly maintained at a lower level at 5mM K, the authors should ideally perform a time course showing that at short, medium and long timepoint the effect of WNK1 knockout on phosphorylation of SGK1 is maintained.
2. Could the authors explain better why 5mM K+ reduces PSPAK levels?
3. In fig.4A the authors reconstitute the WNK1 knockout cells with various forms of wild type and kinase inactive WNK1. It would be important that the authors show the control blots monitoring the levels of the different WNK isoforms in reconstituted cells, compared to that of the endogenous WNK1 shown in the WT cell lines. This should be shown in fig.4.
4. I assume the WNK1 knockout cells are stably reconstituted with WNK1 forms?
5. Similarly, for fig.4C, control immunoblots blots are needed to show relative levels of each of the various reconstituted components that are employed in this experiment.
6. The effect of the kinase dead WNK1 reconstituting the loss of WNK1 is only shown using the short 1-491 form of WNK1. Ideally the authors should demonstrate that the long WNK1 kinase dead mutant was also able to reconstitute SGK1 phosphorylation.
7. In fig.4B that authors also demonstrate that the phosphorylation of SPAK and OSR1 seems to be restored with a kinase dead WNK1 1-491 construct. This is a surprising result, as for SPAK and OSR1 phosphorylation presumably requires WNK1 mediated phosphorylation. There are multiple WNK isoforms in cells and one could imagine that potentially over-expression of the kinase inactive 1-491 WNK1 could somehow interact with WNK2, WNK3 or WNK4 in cells and stimulate the activity of these other WNK isoforms to promote phosphorylation of SPAK and OSR1. If this is happening, this could also explain why SGK1 phosphorylation is reconstituted with the kinase inactive WNK1.
8. For the reconstitution experiments shown in fig.6C control blots showing levels of WNK1, SIN1, SGK1 in the reconstitution experiments at the different doses of [Cl-]i should be shown.
9. In fig.6C, what is the purpose of the right-hand panel blot, showing data with the wild type HEK293 cells? Ideally shouldn't this be run in the same experiment as the WNK1 knockout cells shown on the left hand panel? Why is quantitation undertaken for the right-hand panel of fig.4C but not the left-hand panel?

<u>Reviewer 2</u>

Advance summary and potential significance to field

In this study, the authors continue their previous studies of the mTOR signaling and potassium homeostasis in the kidneys. This manuscript demonstrated that WNK1 acts as a scaffold to recruit SGK1 to mTORC2 and enhance K+-stimulated SGK1 HM phosphorylation independent of WNK1 kinase activity in-vitro. In general, this is a well done cell culture study, which provides important information about the studied signaling pathway. However, there are some suggestions/concerns which should be addressed:

Comments for the author

1. The authors should attempt to confirm the key observation(s) of their study in one of the animal models to extend their research from cell culture to more physiological systems. The authors should be able to perform these experiments considering the availability of various animal models and all tools such as abs, etc.

© 2022. Published by The Company of Biologists under the terms of the Creative Commons Attribution License (https://creativecommons.org/licenses/by/4.0/).

2. The authors should explain why kinase-dead WNK1 and L-WNK1 showed a similar effect on K+-induced ENaC current while WNK inhibitor (WNK643) significantly impaired the ENaC current at 1 µM.
3. The authors should provide the quantification of pSPAK/tSPAK ratio of the WNK1-/- cells transfected with different types of WNK1 to prove the kinase activity.
4. The authors have used different concentrations of Cl- in WNK1-/- cells (Figures 2 and 3). What is the rationale for choosing these 2 concentrations?
There is no description in both figure legends and results. Also, the authors should indicate the Cl- concentrations in WT cells and use the same concentrations of Cl- consistent with WNK1-/- cells.
5. Figure 1C: Authors should provide the quantification of the SGK1 phosphorylation level (pSGK1/tSGK1), from the blot itself, it's hard to interpret the "marked decrease of SGK1 Ser-422 phosphorylation in mpkCCDRictor-/- cells" as described in the manuscript. The authors should also describe which antibody they used and the expected molecular weight since (1) two pSGK1(S422) antibodies were listed in Table S1 and (2) Figure 1C showed multiple bands around 50 kDa.
6. Figures 2B: The authors should describe how did they calculate pA. What is "multi-channel" cell-attached configuration? The authors should also provide the representative recordings and I/V. Po and/or NPo should be calculated as well.
7. Figure 4A: The authors should explain why they switched the K+ concentration to 1.5 and 5 mM while previous experiments were performed under 1 and 5 mM.
8. Figure 4C: The authors should provide representative recordings.
9. Please consider discussing the recent studies about WNK-1 control of BK channels in ICs (PMID: 34229479).
10. It is unclear why all figures are black and white, and figure 6 uses red symbols. It should be consistent between the figures.

First revision

Author response to reviewers' comments

Point-by-point Response to Reviewers.

Reviewer 1 Advance Summary and Potential Significance to Field:
The authors of this study investigate the role that the WNK1 kinase may play in controlling the ability of the mTORC2 complex in regulate the phosphorylation and activation of the SGK1 protein kinase. The authors' data suggest that in response to high extracellular K+ and high [Cl-]i that WNK1 and SGK1 are recruited to the mTORC2 complex and that this is required for optimal phosphorylation of SGK1 at its hydrophobic motif. Interestingly the authors' data suggest that the ability of WNK1 to promote mTORC2 mediated activation of SGK1 is not dependent upon its kinase activity. The data are fairly convincing, and the study would make a useful contribution to the literature in this area and potentially stimulate further research.

RESPONSE: We appreciate the overall positive response to our work.

Reviewer 1 Comments for the Author:

1. In fig.3 the authors only study the effect of 5mM K+ at a single timepoint. To demonstrate that SGK1 phosphorylation is constantly maintained at a lower level at 5mM K, the authors should ideally perform a time course showing that at short, medium and long timepoint the effect of WNK1 knockout on phosphorylation of SGK1 is maintained.

RESPONSE:

We have now done time course experiments in WT and WNK1$^{-/-}$ mpkCCD cells in which cells were adapted to 1 mM K+ and then the media K+ concentration was raised to 5 mM, as in Fig. 2C. After 6 and 24 h of incubation in 5 mM K+ containing media, cells were harvested and the protein expressions were analyzed by western blot. In WT cells, pSGK1 remained elevated until at least 24 h However, in WNK 1KO cells pSGK1 level was substantially lower over the

© 2022. Published by The Company of Biologists under the terms of the Creative Commons Attribution License (https://creativecommons.org/licenses/by/4.0/).

course of the experiment. The new data have been added to the supplemental figures (now Fig. S2). Note also that as we have seen in other contexts, the pSGK1 level in WT cells subsides somewhat between 1 and 6 h.

2. *Could the authors explain better why 5mM K+ reduces PSPAK levels?*

RESPONSE: It is well established in multiple cell types that high extracellular K inhibits WNK-dependent SPAK phosphorylation, likely due to depolarization of the cells and increased intracellular Cl- concentration. In mpkCCD cells we observed similar results; we believe that this is the first demonstration of this effect in mpkCCD cells.

3. *In fig.4A the authors reconstitute the WNK1 knockout cells with various forms of wild type and kinase inactive WNK1. It would be important that the authors show the control blots monitoring the levels of the different WNK isoforms in reconstituted cells, compared to that of the endogenous WNK1 shown in the WT cell lines. This should be shown in fig.4.*

RESPONSE: We have added the control blots showing the expression levels of different WNK isoforms to old Fig 4B (now Fig. 3B). The western blot data suggests that expression levels of the shorter forms of WNK1 (1-491) were slightly higher in the transfected cells as compared to the endogenous WNK1 in the WT cells. The expression level of the full length WNK1 in the transfected cells was comparable with the expression of the endogenous WNK1 in the WT mpkCCD cells. Transfected WNK1 expression was not affected by changes in extracellular [K+].

4. *I assume the WNK1 knockout cells are stably reconstituted with WNK1 forms?*

RESPONSE: The WNK1 knockout cells were transiently transfected with WNK1 isoforms. However, in parallel experiments we co-transfected the cells with GFP along with the WNK1 isoforms and monitored their transfection efficiency under fluorescence microscope, which showed about 90% cells were transfected with the expression constructs. We also verified that the overall expression of WNK was not markedly different from endogenous.

5. *Similarly, for fig.4C, control immunoblots blots are needed to show relative levels of each of the various reconstituted components that are employed in this experiment.*

RESPONSE: Control immunoblots for various expression constructs have been added to the supplemental figures (now Fig. S3).

6. *The effect of the kinase dead WNK1 reconstituting the loss of WNK1 is only shown using the short 1-491 form of WNK1. Ideally the authors should demonstrate that the long WNK1 kinase dead mutant was also able to reconstitute SGK1 phosphorylation.*

RESPONSE: We have now performed additional experiments using L-WNK1 (WT and Kinase-dead). The observed effects were similar to those of the truncated variants. The new data is added to the main figures (now Fig. 4B).

7. *In fig.4B that authors also demonstrate that the phosphorylation of SPAK and OSR1 seems to be restored with a kinase dead WNK1 1-491 construct. This is a surprising result, as for SPAK and OSR1 phosphorylation presumably requires WNK1 mediated phosphorylation. There are multiple WNK isoforms in cells and one could imagine that potentially over-expression of the kinase inactive 1-491 WNK1 could somehow interact with WNK2, WNK3 or WNK4 in cells and stimulate the activity of these other WNK isoforms to promote phosphorylation of SPAK and OSR1. If this is happening, this could also explain why SGK1 phosphorylation is reconstituted with the kinase inactive WNK1.*

RESPONSE: The reviewer raises an important point: we are not able to say that no WNK kinase activity is implicated in our results. Other WNK kinases are still expressed in the WNK1 KO mpkCCD cells, notably WNK4. We attempted unsuccessfully to delete WNK4. Prior publications have shown interactions of WNK1 and WNK4, and it is possible that the kinase activity of WNK4 (or conceivably another WNK such as WNK3) is implicated. Along these lines, it is further notable that the WNK kinase inhibitor WNK-463 did, at high concentrations, inhibit mTORC2 phosphorylation of SGK1. The

© 2022. Published by The Company of Biologists under the terms of the Creative Commons Attribution License (https://creativecommons.org/licenses/by/4.0/).

concentrations required were 10-fold higher than those inhibiting WNK-dependent SPAK phosphorylation and hence this effect could be an off-target one, but we cannot say that with confidence. It is important to note that our primary thesis is that WNK1, independent of its kinase activity, is serving as a scaffold that brings together SGK1 and mTORC2 to enhance SGK1 phosphorylation. We think that this important point remains intact, however, we have modified the text to take into account the possible catalytic role of another WNK.

8. For the reconstitution experiments shown in fig.6C control blots showing levels of WNK1, SIN1, SGK1 in the reconstitution experiments at the different doses of [Cl-]i should be shown.

RESPONSE: We have added the control blots for WNK1 (Myc), SIN1 (V5) and tSGK1 for these experiments to the figures (now Fig. S6B) as suggested by the Reviewer. The data showed that there was no effect of changing Cl- concentrations on the expression levels of WNK1 and Sin1. In light of new data in WT cells the reconstitution experiment has been moved to supplementary figures.

9. In fig.6C, what is the purpose of the right-hand panel blot, showing data with the wild type HEK293 cells? Ideally shouldn't this be run in the same experiment as the WNK1 knockout cells shown on the left hand panel?

RESPONSE: We apologize for the lack of clarity here. Old Fig. 6 has been extensively reworked based on additional experiments and reorganization of the figure for clarity. Please see new Fig. 6C and 6D, and associated text. These experiments were performed in WT cells.

We also performed experiments over a wider range of Cl concentrations in WNK1 KO-HEK293 cells which had been transfected with WT WNK1. As noted above, this figure has been moved to supplementary figures (now Fig. S6B).

Why is quantitation undertaken for the right-hand panel of fig.4C but not the left-hand panel?

RESPONSE: We assume the reviewer means Fig. 6C here, not 4C. During the initial submission of our manuscript, we did not have enough repeats of the experiment in left-hand panel of original Fig. 6C, which showing the Cl- dependence in WT HEK-293 cells. We now have sufficient repeats of the experiment to perform statistical analysis, shown in Fig. S6C.

Reviewer 2 Advance Summary and Potential Significance to Field:

In this study, the authors continue their previous studies of the mTOR signaling and potassium homeostasis in the kidneys. This manuscript demonstrated that WNK1 acts as a scaffold to recruit SGK1 to mTORC2 and enhance K+-stimulated SGK1 HM phosphorylation independent of WNK1 kinase activity in-vitro. In general, this is a well done cell culture study, which provides important information about the studied signaling pathway. However, there are some suggestions/concerns which should be addressed:

RESPONSE: We appreciate the reviewer's overall positive response, and address below the suggestions/concerns.

Reviewer 2 Comments for the Author:

1. The authors should attempt to confirm the key observation(s) of their study in one of the animal models to extend their research from cell culture to more physiological systems. The authors should be able to perform these experiments considering the availability of various animal models and all tools such as abs, etc.

RESPONSE: We appreciate these suggestions and agree that experiments using animal models would help solidify the physiological relevance of our study. However, we believe that this is beyond the scope of the current work, which focuses predominantly on the underlying cellular mechanism for direct effects of K+ in principal cells. The only animal model which is truly

© 2022. Published by The Company of Biologists under the terms of the Creative Commons Attribution License (https://creativecommons.org/licenses/by/4.0/).

relevant is a tubule-specific WNK1 KO mouse, which is not currently available. Also, given the marked effects of WNK1 and/or WNK4 in the DCT, we would need a segment-specific KO. The mpkCCD cells are a well validated model for principal cells, and we believe an ideal model for the present mechanistic study. We have extensively characterized the role of WNK1 in regulating mTORC2 using this system, including CRISPR-mediated KO of both WNK1 and Rictor, and have significantly extended our earlier study which suggested but did not demonstrate a role for WNK1 in mediating local effects of K+ on ENaC via mTORC2.

2. The authors should explain why kinase-dead WNK1 and L-WNK1 showed a similar effect on K+-induced ENaC current while WNK inhibitor (WNK643) significantly impaired the ENaC current at 1 μM.

RESPONSE: This raises a very important point, which we had not adequately addressed in the original manuscript. Please also see response to Reviewer 1 point 7. We had interpreted the effect of the WNK kinase inhibitor WNK463 on pSGK1 and ENaC as an off-target effect. It occurs at 10-fold greater concentration than needed for inhibition of pSPAK which, together with other corroborating data (eg, the effect of kinase-dead WNK1 on interaction of SGK1 with mTORC2) contributed to our impression that the mechanism did not involve the kinase activity of any WNK. However, we do not have direct evidence establishing that. It is certainly possible that the kinase activity of another WNK, eg WNK4, is important for the effects we see. It is important to emphasize, however, that the fundamental novel point of our paper is that WNK1 acts through a non-catalytic (likely scaffolding) mechanism to selectively modulate mTORC2 phosphorylation of SGK1 (and not Akt), and thereby mediate [K+] stimulation of ENaC. We have clarified in the text that it remains possible that the kinase activity of another WNK may be implicated.

3. The authors should provide the quantification of pSPAK/tSPAK ratio of the WNK1-/- cells transfected with different types of WNK1 to prove the kinase activity.

RESPONSE: The quantification of pSPAK/tSPAK was added to the main Figure (now Fig. 3C, lower panel).

4. The authors have used different concentrations of Cl- in WNK1-/- cells (Figures 2 and 3). What is the rationale for choosing these 2 concentrations? There is no description in both figure legends and results. Also, the authors should indicate the Cl- concentrations in WT cells and use the same concentrations of Cl- consistent with WNK1-/- cells.

RESPONSE: We apologize for this confusion. In Figures 2 and 3, 'Cl' was the abbreviation for 'clone' and not Chloride. We have made changes to the labeling and represented them as 'clone' to make it clear to readers.

5. Figure 1C: Authors should provide the quantification of the SGK1 phosphorylation level pSGK1/tSGK1), from the blot itself, it's hard to interpret the "marked decrease of SGK1 Ser-422 phosphorylation in mpkCCDRictor-/- cells" as described in the manuscript.

RESPONSE: We have added the quantification data of pSGK1 for Fig. 1C as suggested by the Reviewer. The new data is presented as Fig. 1C, lower panel.

The authors should also describe which antibody they used and the expected molecular weight since (1) two pSGK1(S422) antibodies were listed in Table S1 and (2) Figure 1C showed multiple bands around 50 kDa.

RESPONSE: We have added the information about the antibodies used for that experiment, to the Figure legend (Figure 1C). The phosphorylation of SGK1 can be detected as multiple bands (around 55 KDa) as SGK1 undergoes multiple phosphorylations (at least 3 are best known as of now) which leads to MW changes in the pSGK1 bands depending on the phosphorylation status of SGK1 in the cells. Further, SGK1 has multiple translation start sites, which give different size proteins.

© 2022. Published by The Company of Biologists under the terms of the Creative Commons Attribution License (https://creativecommons.org/licenses/by/4.0/).

6. Figures 2B: The authors should describe how did they calculate pA. What is "multi-channel" cell attached configuration? The authors should also provide the representative recordings and I/V. Po and/or NPo should be calculated as well.

RESPONSE: pA was measured in cell-attached mode. This has been clarified in the Methods. In multichannel cell attached mode net current (in pA) is measured in individual patches at specific holding potentials, as shown in the I/V curve in Fig. S1. The measured pA signal in this case reflects the sum of all the channel activities in one patch. Signal to noise ratio was not sufficient to separately calculate N and Po, however, we believe that this patch clamp data still provides a helpful complement to the transepithelial currents shown in Fig. 2B. These latter measurements were performed using mpkCCD cells grown on Transwell filters, and reflect net amiloride-sensitive Na+ currents.

7. Figure 4A: The authors should explain why they switched the K+ concentration to 1.5 and 5 mM while previous experiments were performed under 1 and 5 mM.

RESPONSE: We have extensively characterized the mTORC2 and ENaC activity under a variety of extracellular [K+] conditions in this and prior studies. Initially for the present study, we used 1 mM [K+] as a baseline in order maximize the separation between low and high K+ conditions. However, we found later that 1.5 mM gave a similar baseline, and we chose to use that concentration which is (slightly) more physiological.

8. Figure 4C: The authors should provide representative recordings.

RESPONSE: We have added this data to the supplemental figures (now Fig. S4).

9. Please consider discussing the recent studies about WNK-1 control of BK channels in ICs (PMID: 34229479).

RESPONSE: Thank you for pointing this out. We have added that reference to the discussion of our revised manuscript.

10. It is unclear why all figures are black and white, and figure 6 uses red symbols. It should be consistent between the figures.

RESPONSE: We have changed the data points from closed red circles to black and white open circles for consistency with other figures.

Second decision letter

MS ID#: JOCES/2022/260313

MS TITLE: WNK1 is a chloride-stimulated scaffold that regulates mTORC2-activity and ion transport

AUTHORS: Bidisha Saha, Deise Carla Almeida Leite Dellova, John Demko, Mads Vaarby Sorensen, Enzo Takagi, Catherine Gleason, Waheed Shabbir, and David Pearce
ARTICLE TYPE: Research Article

I am happy to tell you that your manuscript has been accepted for publication in Journal of Cell Science, pending standard ethics checks.

Reviewer 1

Advance summary and potential significance to field

The authors have carefully revised their manuscript to address the points that the Reviewers have made. The revisions are adequate in my opinion and I am pleased to recommend acceptance of the manuscript

© 2022. Published by The Company of Biologists under the terms of the Creative Commons Attribution License (https://creativecommons.org/licenses/by/4.0/).

Comments for the author

see above

Reviewer 2

Advance summary and potential significance to field

The authors addressed most of the raised questions. My only concern left is related to electrophysiology and is summarized below.

Comments for the author

Data shown in the new supplementary Figure 1 still do not include representative recordings as requested. Representative currents reported in Figure S4 have a high signal-to-noise ratio and drifting baseline. Thus, the calculation of NPo based on these recordings should be problematic. It is suggested to either provide better quality representative currents (for both figure S1 and Figure 4) or remove all patch-clamp data.

© 2022. Published by The Company of Biologists under the terms of the Creative Commons Attribution License (https://creativecommons.org/licenses/by/4.0/).
